# Author Correction: Early-childhood linear growth faltering in low- and middle-income countries

**DOI:** 10.1038/s41586-024-08344-6

**Published:** 2024-12-12

**Authors:** Jade Benjamin-Chung, Andrew Mertens, John M. Colford, Alan E. Hubbard, Mark J. van der Laan, Jeremy Coyle, Oleg Sofrygin, Wilson Cai, Anna Nguyen, Nolan N. Pokpongkiat, Stephanie Djajadi, Anmol Seth, Wendy Jilek, Esther Jung, Esther O. Chung, Sonali Rosete, Nima Hejazi, Ivana Malenica, Haodong Li, Ryan Hafen, Vishak Subramoney, Jonas Häggström, Thea Norman, Kenneth H. Brown, Parul Christian, Benjamin F. Arnold, The Ki Child Growth Consortium, Souheila Abbeddou, Linda S. Adair, Tahmeed Ahmed, Asad Ali, Hasmot Ali, Per Ashorn, Rajiv Bahl, Mauricio L. Barreto, France Begín, Pascal Obong Bessong, Maharaj Kishan Bhan, Nita Bhandari, Santosh K. Bhargava, Zulfiqar A. Bhutta, Robert E. Black, Ladaporn Bodhidatta, Delia Carba, Ines Gonzalez Casanova, William Checkley, Jean E. Crabtree, Kathryn G. Dewey, Christopher P. Duggan, Caroline H. D. Fall, Abu Syed Golam Faruque, Wafaie W. Fawzi, José Quirino da Silva Filho, Robert H. Gilman, Richard L. Guerrant, Rashidul Haque, Sonja Y. Hess, Eric R. Houpt, Jean H. Humphrey, Najeeha Talat Iqbal, Elizabeth Yakes Jimenez, Jacob John, Sushil Matthew John, Gagandeep Kang, Margaret Kosek, Michael S. Kramer, Alain Labrique, Nanette R. Lee, Aldo Ângelo Moreira Lima, Mustafa Mahfuz, Tjale Cloupas Mahopo, Kenneth Maleta, Dharma S. Manandhar, Karim P. Manji, Reynaldo Martorell, Sarmila Mazumder, Estomih Mduma, Venkata Raghava Mohan, Sophie E. Moore, Ishita Mostafa, Robert Ntozini, Mzwakhe Emanuel Nyathi, Maribel Paredes Olortegui, William A. Petri, Prasanna Samuel Premkumar, Andrew M. Prentice, Najeeb Rahman, Harshpal Singh Sachdev, Kamran Sadiq, Rajiv Sarkar, Naomi M. Saville, Saijuddin Shaikh, Bhim P. Shrestha, Sanjaya Kumar Shrestha, Alberto Melo Soares, Bakary Sonko, Aryeh D. Stein, Erling Svensen, Sana Syed, Fayaz Umrani, Honorine D. Ward, Keith P. West, Lee Shu Fune Wu, Seungmi Yang, Pablo Penataro Yori

**Affiliations:** 1 Department of Epidemiology & Population Health, Stanford University, Stanford, CA USA; 2 Division of Epidemiology & Biostatistics, University of California, Berkeley, Berkeley, CA USA; 3 Chan Zuckerberg Biohub, San Francisco, CA USA; 4 Hafen Consulting, LLC, West Richland, WA USA; 5 DVPL Tech, Dubai, United Arab Emirates; 6 Cytel Inc., Waltham, MA USA; 7 Quantitative Sciences, Bill & Melinda Gates Foundation, Seattle, WA USA; 8 Department of Nutrition, University of California, Davis, Davis, CA USA; 9 Center for Human Nutrition, Department of International Health, Johns Hopkins Bloomberg School of Public Health, Baltimore, MD USA; 10 Francis I. Proctor Foundation, University of California, San Francisco, San Francisco, CA USA; 11 Department of Ophthalmology, University of California, San Francisco, San Francisco, CA USA; 12 Department of Public Health and Primary Care, Ghent University, Ghent, Belgium; 13 Department of Nutrition, University of North Carolina at Chapel Hill, Chapel Hill, NC USA; 14 International Centre for Diarrhoeal Disease Research, Bangladesh, Dhaka, Bangladesh; 15 Department of Pediatrics and Child Health, The Aga Khan University, Karachi, Pakistan; 16 JiVitA Project, John Hopkins Bangladesh, Rangpur, Bangladesh; 17 Center for Child, Adolescent, and Maternal Health Research, Department of Medicine and Health Technology, Tampere University, Tampere, Finland; 18 Tampere University Hospital, Tampere, Finland; 19 World Health Organization, Geneva, Switzerland; 20 Center of Data and Knowledge Integration for Health, Fundação Oswaldo Cruz, Salvador, Brazil; 21 UNICEF, New York, NY USA; 22 HIV/AIDS & Global Health Research Programme, University of Venda, Thohoyandou, South Africa; 23 Indian Institute of Technology, New Delhi, India; 24 Centre for Health Research and Development, Society for Applied Studies, New Delhi, India; 25 Sunder Lal Jain Hospital, New Delhi, India; 26 Centre of Excellence in Women & Child Health, Institute for Global Health & Development, The Aga Khan University, Karachi, Pakistan; 27 Johns Hopkins University Bloomberg School of Public Health, Baltimore, MD USA; 28 Armed Forces Research Institute of Medical Sciences, Bangkok, Thailand; 29 USC Office of Population Studies Foundation Inc., University of San Carlos, Cebu, The Philippines; 30 Rollins School of Public Health, Emory University, Atlanta, GA USA; 31 Leeds Institute for Medical Research, St. James’s University Hospital, University of Leeds, Leeds, UK; 32 Department of Nutrition and Institute for Global Nutrition, University of California Davis, Davis, CA USA; 33 Center for Nutrition, Boston Children’s Hospital, Boston, MA USA; 34 MRC Lifecourse Epidemiology Centre, University of Southampton, Southampton, UK; 35 Department of Global Health and Population, Harvard TH Chan School of Public Health, Boston, MA USA; 36 Federal University of Ceará, Fortaleza, Brazil; 37 University of Virginia, Charlottesville, VA USA; 38 Department of Pediatrics, University of New Mexico Health Sciences Center, Albuquerque, NM USA; 39 Department of Internal Medicine, University of New Mexico Health Sciences Center, Albuquerque, NM USA; 40 Christian Medical College, Vellore, India; 41 Low Cost Effective Care Unit, Christian Medical College, Vellore, India; 42 Translational Health Science and Technology Institute, Faridabad, India; 43 McGill University, Montreal, Quebec Canada; 44 McGill University Health Centre, Montreal, Quebec Canada; 45 Department of Nutrition, School of Health Sciences, University of Venda, Thohoyandou, South Africa; 46 School of Public Health and Family Medicine, College of Medicine, University of Malawi, Zomba, Malawi; 47 Mother and Infant Research Activities, Kathmandu, Nepal; 48 Department of Pediatrics and Child Health, Muhimbili University School of Health and Allied Sciences, Dar es Salaam, Tanzania; 49 Haydom Lutheran Hospital, Haydom, Tanzania; 50 Department of Women and Children’s Health, King’s College London, London, UK; 51 MRC Unit The Gambia at London School of Hygiene and Tropical Medicine, Banjul, The Gambia; 52 Zvitambo Institute for Maternal and Child Health Research, Harare, Zimbabwe; 53 Department of Animal Sciences, School of Agriculture, University of Venda, Thohoyandou, South Africa; 54 AB PRISMA, Lima, Peru; 55 Department of Pediatrics and Clinical Epidemiology, Sitaram Bhartia Institute of Science and Research, New Delhi, India; 56 Institute for Global Health, University College London, London, UK; 57 Health Research and Development Forum, Kathmandu, Nepal; 58 Walter Reed/AFRIMS Research Unit, Kathmandu, Nepal; 59 Haukeland University Hospital, Bergen, Norway; 60 Tufts Medical Center, Tufts University School of Medicine, Boston, MA USA

**Keywords:** Malnutrition, Epidemiology, Paediatric research, Developing world

Correction to: *Nature* 10.1038/s41586-023-06418-5 Published online 13 September 2023

The code that we used to fit meta-analyses for the proportion of children stunted by age in Fig. 3a and Extended Data Figs. 8–10 incorrectly used the total number of children in the denominator for incidence, which incorrectly included children no longer at risk of stunting (i.e., children who had become stunted at the previous age). In the “Onset of stunting in early life” section, in the text now reading “The percentage that experienced incident stunting onset between birth and 3 months ranged from 7% to 57% in each cohort and was 18% overall”, the percentages previously read “6%”, “47%” and “16%.”

We also made analogous corrections in the Supplementary information to figures of age-stratified stunting incidence. Following this correction, age-stratified stunting incidence levels were higher after birth, but trends remained similar overall. We revised the links in the Code availability section for the replication code to include these corrections. Additionally, we mislabeled the *y* axes of Fig. 4b and Extended Data Fig. 12 and their corresponding captions as “Incidence proportion” instead of “Proportion”. We have also corrected a typographical error in cited figures in the Methods “Linear growth velocity” section, and now cite Fig. 6 and Extended Data Fig. 14 in place of Fig. 5 and Extended Data Fig. 10. Additionally, the Reporting summary did not include an email address to contact for data requests, which is now updated. These corrections do not change the inferences we drew from the study. For comparison, original and revised figures are available in the Supplementary information. The updates appear in the HTML and PDF versions of the article.

Supplementary information is available in the online version of this amendment.

## Supplementary information

Original and revised Figs. 3a, 4b, Extended Data Figs. 8, 9, 10, 12

