# Supplementary material for: Early-childhood linear growth faltering in low- and middle-income countries
Source: Nature. 2023 Sep 13;621(7979):550–7. doi: 10.1038/s41586-023-06418-5 (PMC10511325; doi:10.1038/s41586-023-06418-5)
Supplement: Supplementary file 1 — This file contains Supplementary Notes 1–8 and Tables 1–3. [file 41586_2023_6418_MOESM1_ESM.pdf]

nature portfolio

https://doi.org/10.1038/s41586-023-06418-5

Supplementary information

Early-childhood linear growth faltering in low- and middle-income countries

In the format provided by the authors and unedited

Supplementary Information for:

Early childhood linear growth faltering in low- and middle-income countries

Corresponding authors:
Jade Benjamin-Chung, PhD, jadebc@stanford.edu
Benjamin F. Arnold, PhD, ben.arnold@ucsf.edu

Table of Contents

Supplementary Note 1. Severe stunting analyses 2
Supplementary Note 2. Quality assurance........ 3
Supplementary Note 3. Analysis with monthly cohorts 5
3.1 Mean length-for-age Z-score by age........ 5
3.2 Age-specific stunting prevalence 6
3.3 Age-specific stunting incidence........ 6
3.4 Linear growth velocity........ 7
Supplementary Note 4. Assessment of potential secular trends 9
Supplementary Note 5. Analyses of age at first measurement........ 10
5.1 Histogram of first measurement within age 0-30 days 10
5.2 Histogram of age at enrollment........ 11
Supplementary Note 6. Sensitivity analysis using fixed effects........ 12
6.1 Age-specific prevalence 12
6.2 Age-specific incidence........ 13
6.3 Changes in stunting status by age 13
6.4 Linear growth velocity........ 15
Supplementary Note 7. Cohort-specific results 17
7.1 Mean length-for-age Z-score by age........ 17
7.2 Age-specific prevalence 18
7.3 Age-specific incidence........ 20
7.4 Length velocity by age and sex........ 21
7.5 LAZ velocity by age and sex 23
Supplementary Note 8. Primary analyses excluding the PROBIT study 25
8.1 Mean length-for-age Z-score by age........ 25
8.2 Age-specific prevalence 26
8.3 Age-specific incidence........ 26
Supplementary Table 1. Summary of *ki* cohorts........ 28
Supplementary Table 2. Characteristics of participants in each cohort........ 32
Supplementary Table 3. Number of children included by subgroup and region... 35

Supplementary Note 1. Severe stunting analyses

Below, we display plots for the age-specific prevalence and cumulative incidence of severe stunting (LAZ < -3). Overall, the patterns are the same as for stunting (LAZ < -2), with the peak in prevalence at ages 18-24 months and the highest incidence proportion from 0-3 months.

1.1 Age-specific severe stunting prevalence

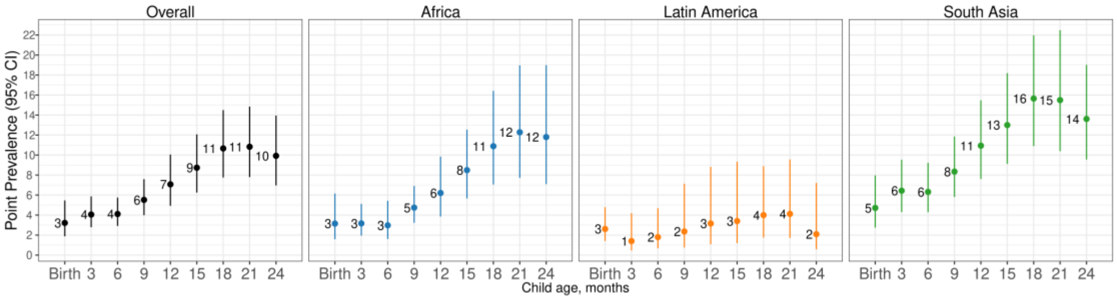

1.2 Age-specific severe stunting incidence

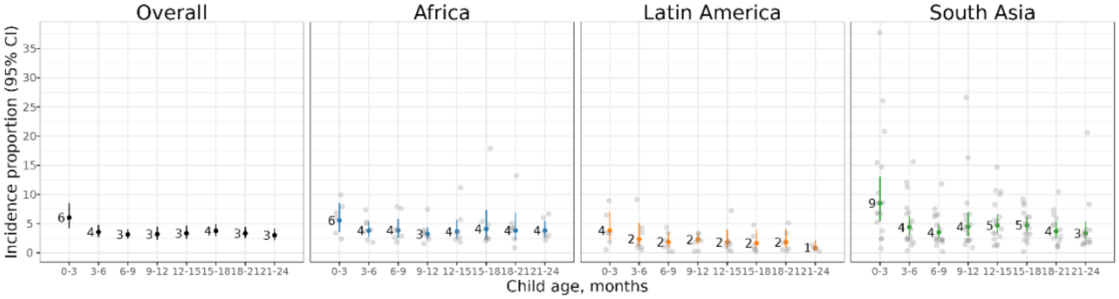

Supplementary Note 2. Quality assurance

To check for outliers in length measurements, we plotted the distribution of raw length measurements by age and sex against different percentiles of the World Health Organization child growth standard distribution. The majority of observations fell within 3 standard deviations of the mean of the standard for males and females. There were certain cohorts, such as the Keneba cohort, that had a larger proportion of observations outside of 3 standard deviations from the mean. There was a higher variability of length measurements in the PROBIT, ZVITAMBO, and Keneba cohorts.

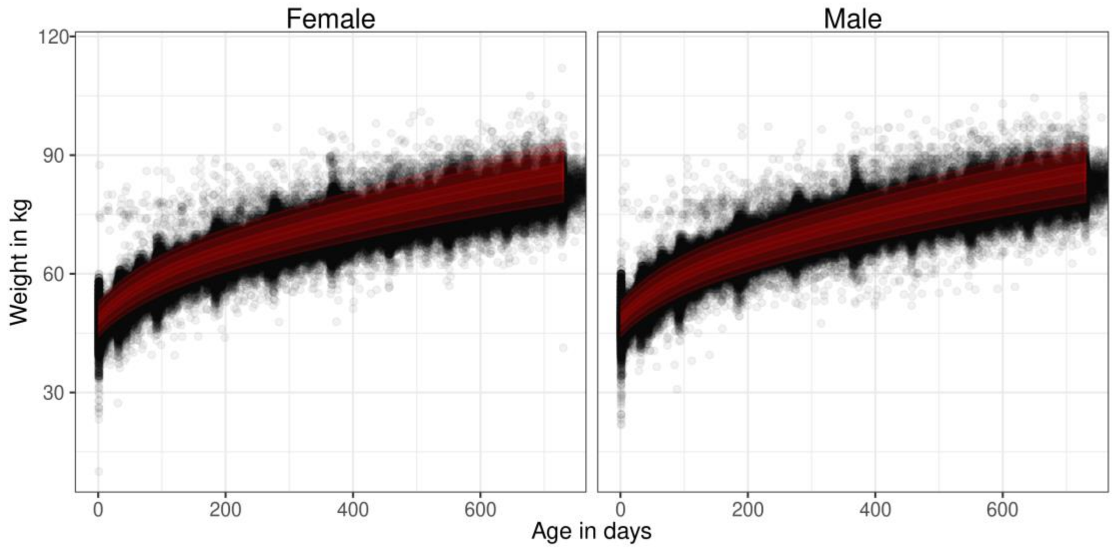

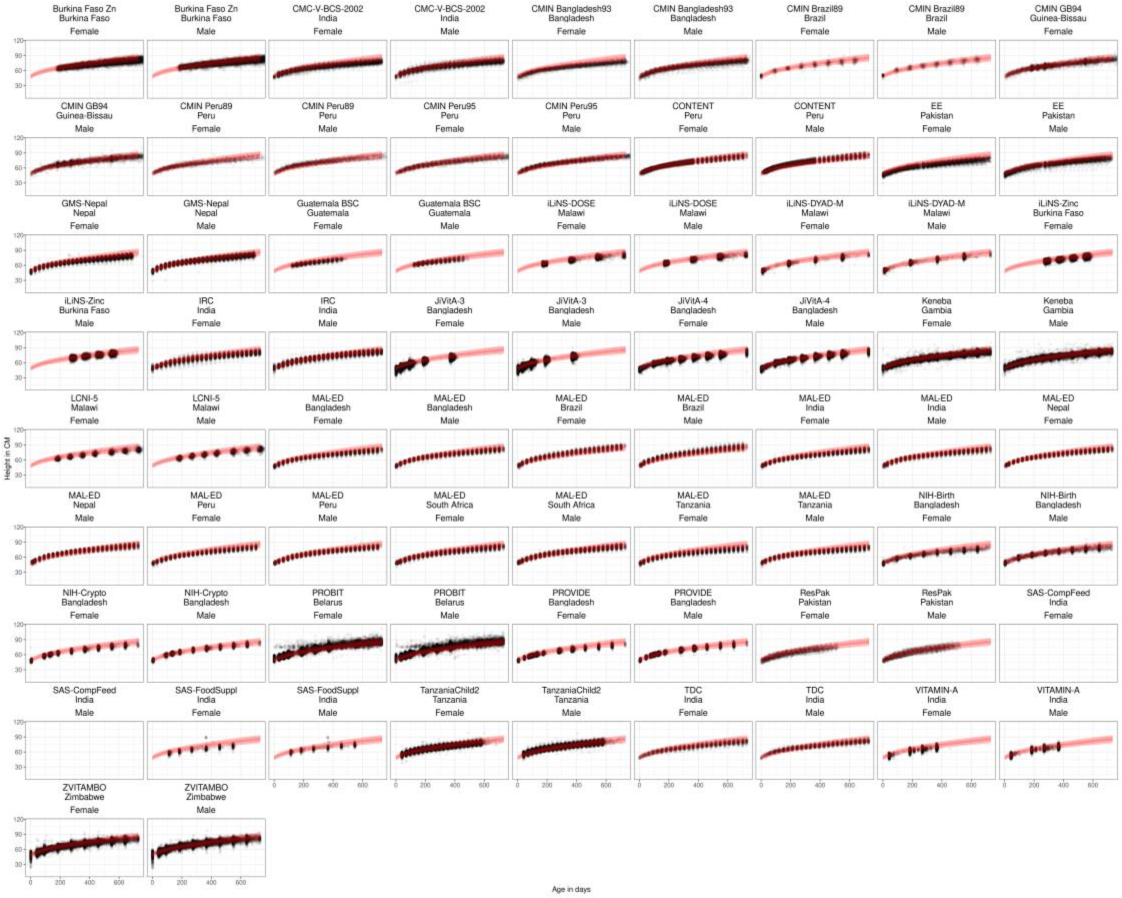
Burkina Faso Zn
Burkina Faso
Female
Burkina Faso Zn
Burkina Faso
Male
CMC-V-BCS-2002
India
Female
CMC-V-BCS-2002
India
Male
CMIN Bangladesh93
Bangladesh
Female
CMIN Bangladesh93
Bangladesh
Male
CMIN Brazil89
Brazil
Female
CMIN Brazil89
Brazil
Male
CMIN GB94
Guinea-Bissau
Female
CMIN GB94
Guinea-Bissau
Male
CMIN Peru89
Peru
Female
CMIN Peru89
Peru
Male
CMIN Peru95
Peru
Female
CMIN Peru95
Peru
Male
CONTENT
Peru
Female
CONTENT
Peru
Male
EE
Pakistan
Female
EE
Pakistan
Male
GMS-Nepal
Nepal
Female
GMS-Nepal
Nepal
Male
Guatemala BSC
Guatemala
Female
Guatemala BSC
Guatemala
Male
iLiNS-DOSE
Malawi
Female
iLiNS-DOSE
Malawi
Male
iLiNS-DYAD-M
Malawi
Female
iLiNS-DYAD-M
Malawi
Male
iLiNS-Zinc
Burkina Faso
Female
iLiNS-Zinc
Burkina Faso
Male
IRC
India
Female
IRC
India
Male
JiVitA-3
Bangladesh
Female
JiVitA-3
Bangladesh
Male
JiVitA-4
Bangladesh
Female
JiVitA-4
Bangladesh
Male
Keneba
Gambia
Female
Keneba
Gambia
Male
LCNI-5
Malawi
Female
LCNI-5
Malawi
Male
MAL-ED
Bangladesh
Female
MAL-ED
Bangladesh
Male
MAL-ED
Brazil
Female
MAL-ED
Brazil
Male
MAL-ED
India
Female
MAL-ED
India
Male
MAL-ED
Nepal
Female
MAL-ED
Nepal
Male
MAL-ED
Peru
Female
MAL-ED
Peru
Male
MAL-ED
South Africa
Female
MAL-ED
South Africa
Male
MAL-ED
Tanzania
Female
MAL-ED
Tanzania
Male
NIH-Birth
Bangladesh
Female
NIH-Birth
Bangladesh
Male
NIH-Crypto
Bangladesh
Female
NIH-Crypto
Bangladesh
Male
PROBIT
Belarus
Female
PROBIT
Belarus
Male
PROVIDE
Bangladesh
Female
PROVIDE
Bangladesh
Male
ResPak
Pakistan
Female
ResPak
Pakistan
Male
SAS-CompFeed
India
Female
SAS-CompFeed
India
Male
SAS-FoodSuppl
India
Female
SAS-FoodSuppl
India
Male
TanzaniaChild2
Tanzania
Female
TanzaniaChild2
Tanzania
Male
TDC
India
Female
TDC
India
Male
VITAMIN-A
India
Female
VITAMIN-A
India
Male
ZVITAMBO
Zimbabwe
Female
ZVITAMBO
Zimbabwe
Male
Height in CM
Age in days

Supplementary Note 3. Analysis with monthly cohorts

To explore the influence of differing numbers of cohorts contributing data at different ages, we conducted a sensitivity analysis in which we subset data to cohorts that measured anthropometry monthly from birth to 24 months.

In this sensitivity analysis, the mean length-for-age Z-score was higher in Latin America and exhibited less of a downwards trajectory with age. Age-specific stunting prevalence and incidence was slightly lower in Latin America and Asia and slightly higher in Africa. Standard errors were smaller for Latin America because the analyses with monthly cohorts excluded the COHORTS Guatemala study, which had a very high stunting prevalence compared to other Latin American cohorts.

3.1 Mean length-for-age Z-score by age

3.1.1 All eligible cohorts

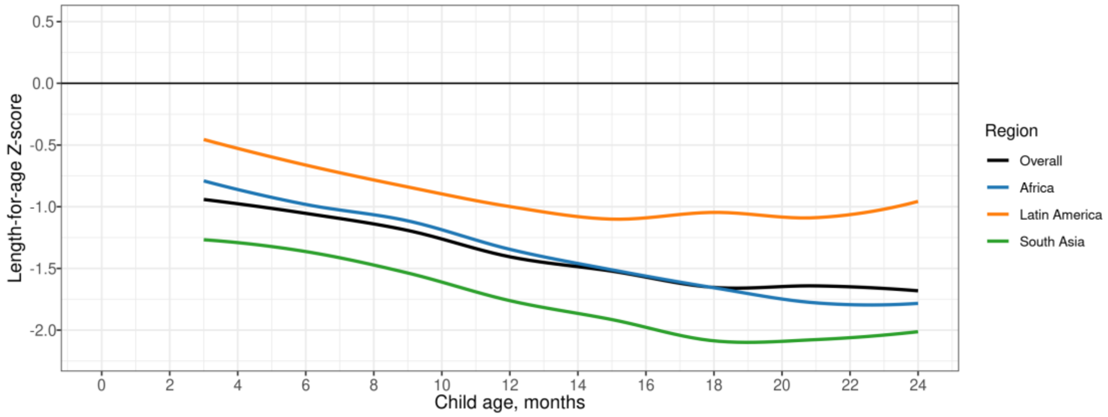

3.1.2 Cohorts that measured monthly from birth to 24 months

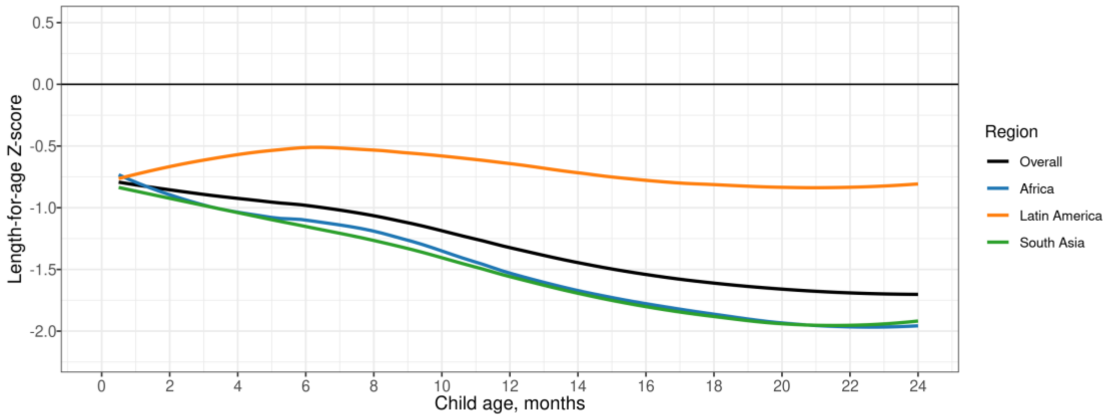

3.2 Age-specific stunting prevalence

3.2.1 All eligible cohorts

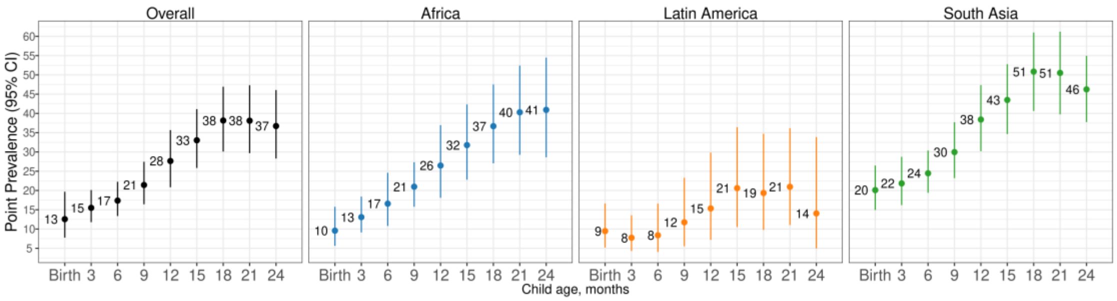

3.2.2 Cohorts that measured monthly from birth to 24 months

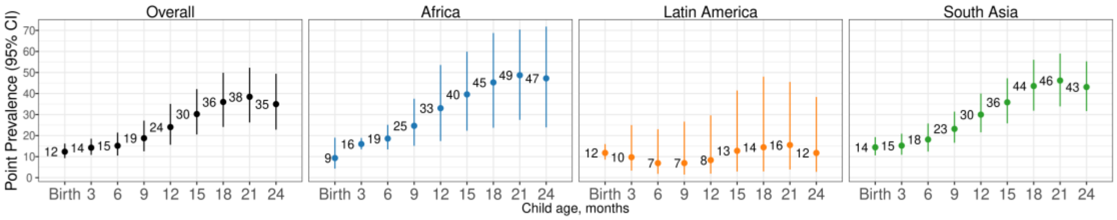

3.3 Age-specific stunting incidence

3.3.1 All eligible cohorts

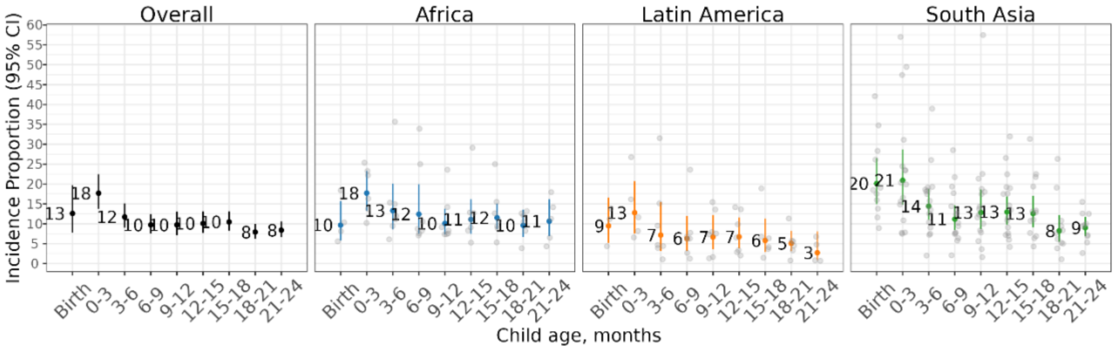

3.3.2 Cohorts that measured monthly from birth to 24 months

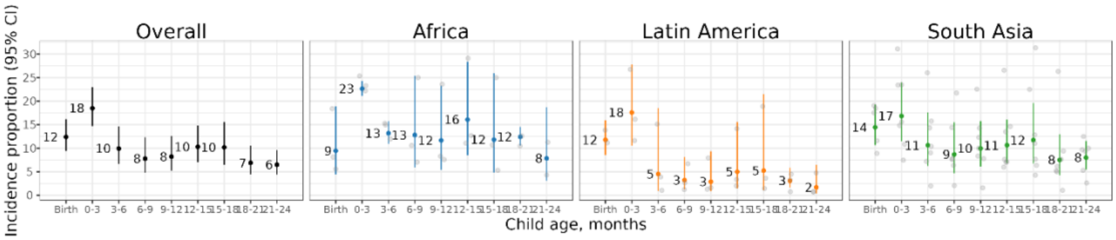

3.4 Linear growth velocity

3.4.1 All eligible cohorts

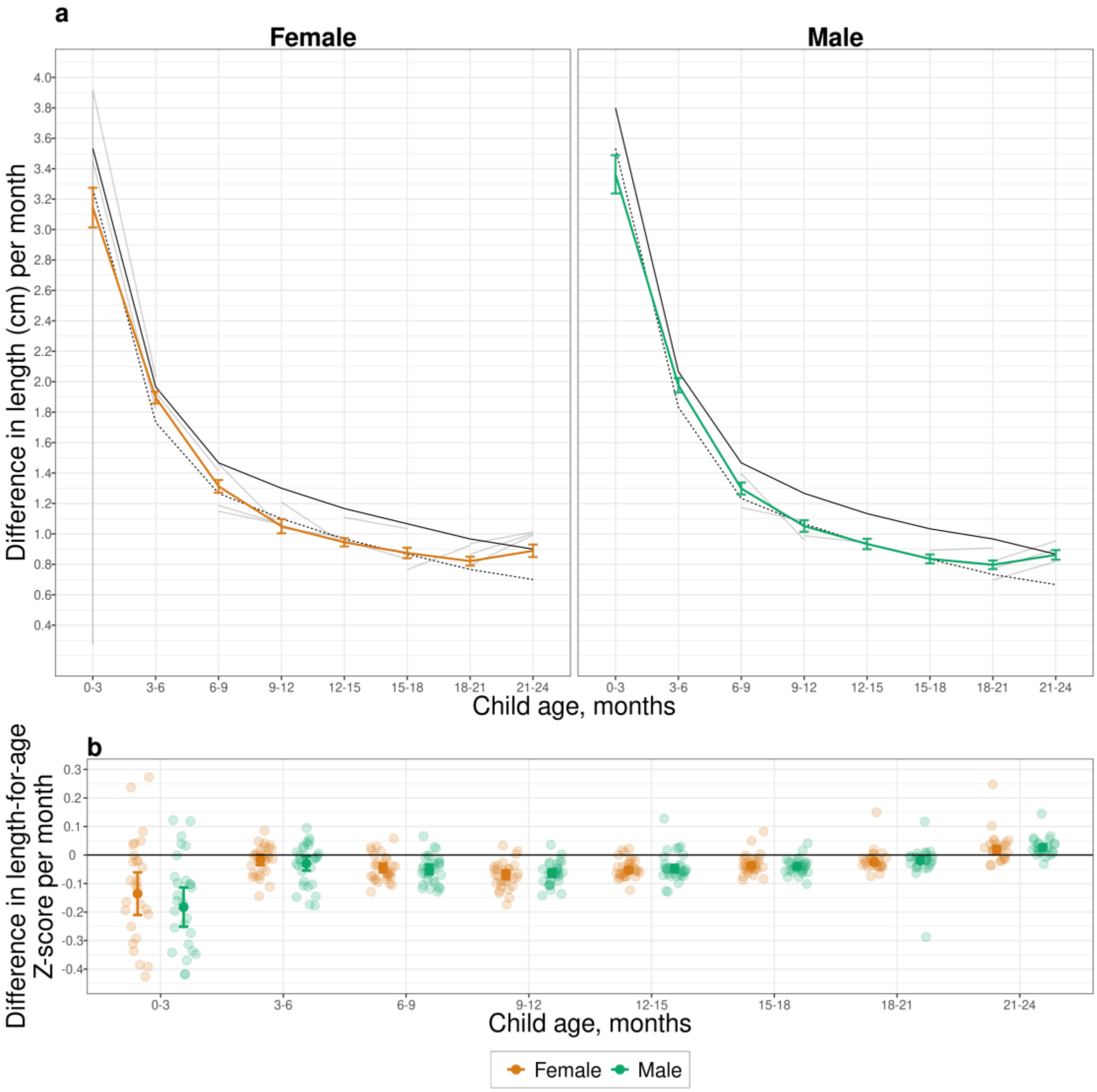

3.4.2 Cohorts that measured monthly from birth to 24 months

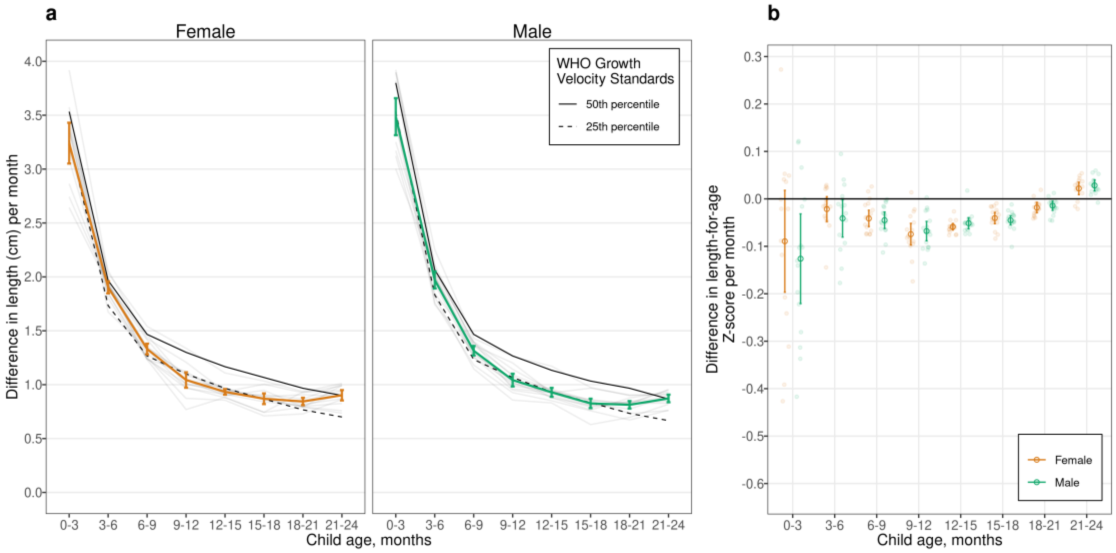

Supplementary Note 4. Assessment of potential secular trends

This study included cohorts that measured child growth from 1987 to 2017. To assess potential secular trends, we plotted the mean length-for-age Z-score (LAZ) over time. The plot below shows the individual observations from included studies over this range of years. Curves were fit to the data using generalized additive models, with the solid line fit through the median LAZ by birth year, and the dashed lines mark the 1st and 3rd quartiles. There does not appear to be a secular trend in LAZ.

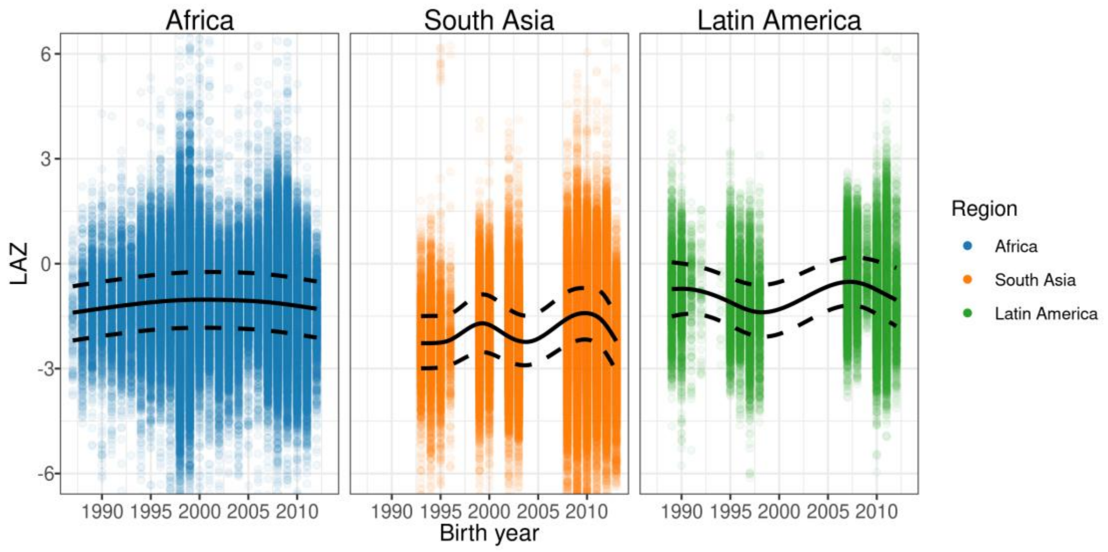

Supplementary Note 5. Analyses of age at first measurement

Our analyses of stunting incidence assumed that children whose first measurement occurred after birth were assumed to have experienced stunting onset at the age halfway between birth and the first measurement. To assess the extent to which this assumption influenced our estimates, we plotted the distribution of age at first measurement and the age at enrollment. The vast majority of children were enrolled close to birth, and the majority were less than 5 days of age at their first measurement.

5.1 Histogram of first measurement within age 0-30 days

5.1.1 All cohorts

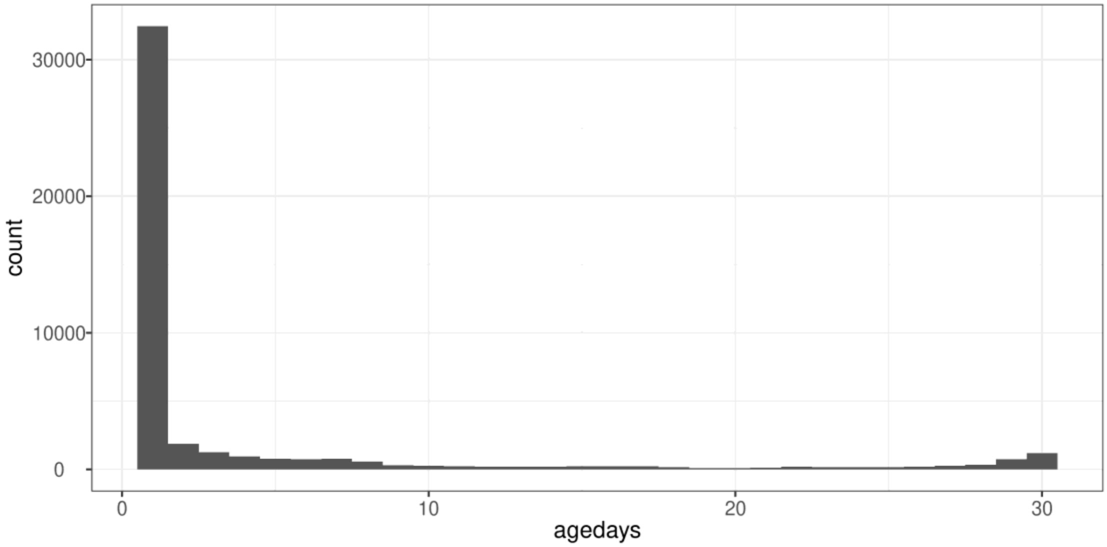

5.1.2 Cohort-stratified

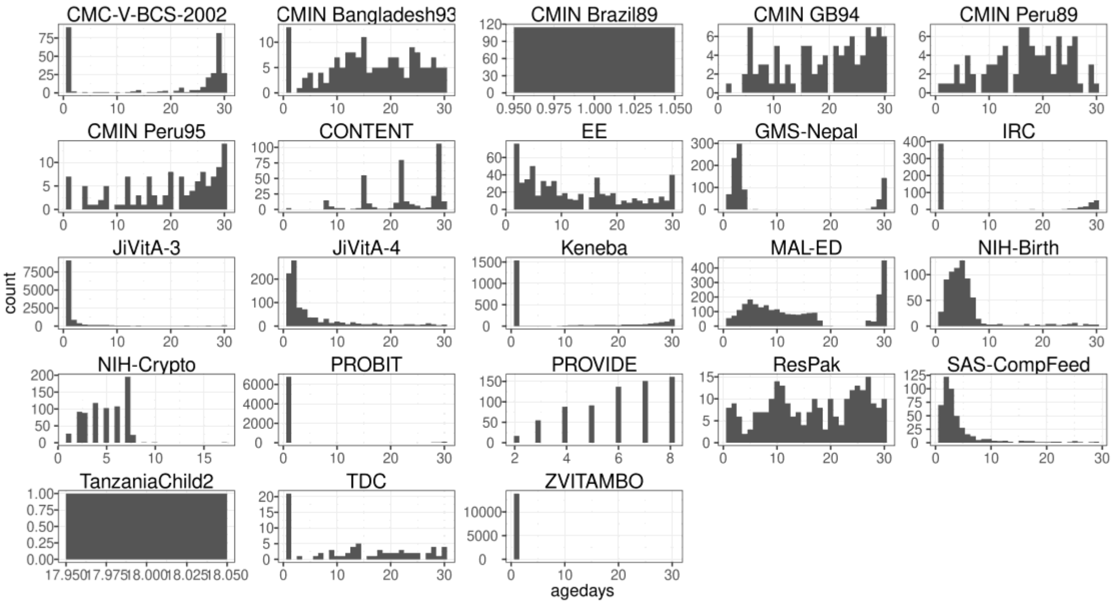

5.2 Histogram of age at enrollment

5.2.1 All cohorts

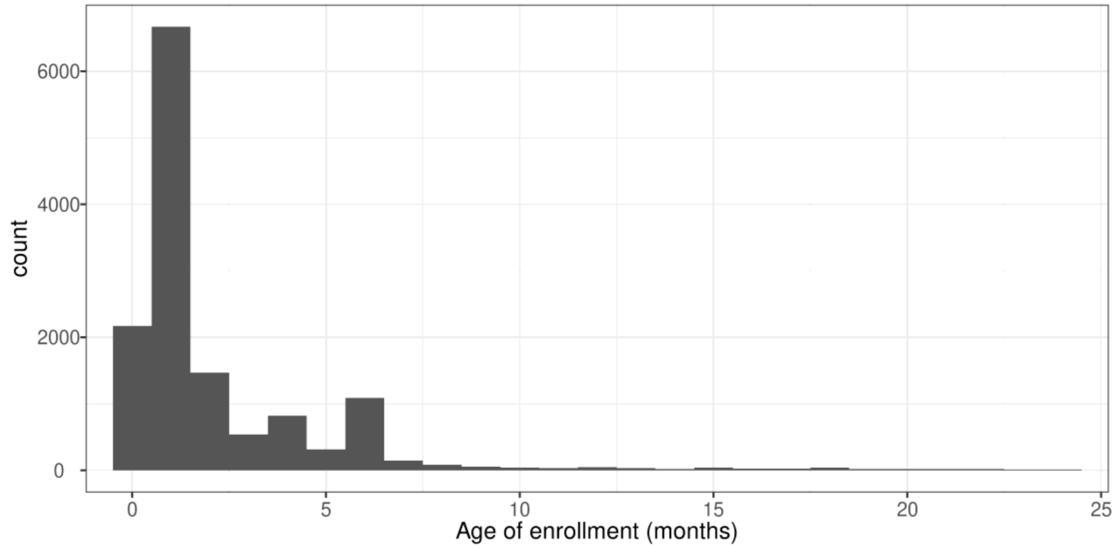

5.2.2 Cohort-stratified

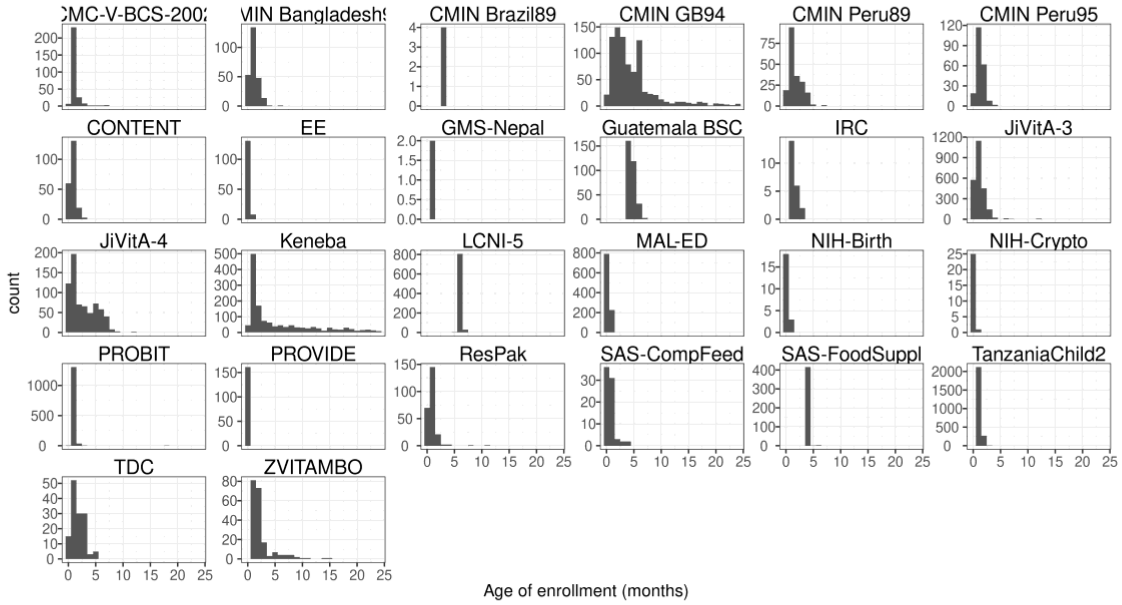

Supplementary Note 6. Sensitivity analysis using fixed effects

The primary analyses presented in this manuscript pooled across individual studies using random effects. Inferences about estimates from fixed effects models are restricted to only the included studies.[1] The random effects approach was more conservative in the presence of study heterogeneity, as evidenced by larger confidence intervals around each point estimates. Overall, the inference from results produced by each method was similar.

The pooled estimates using random effects vs. fixed effects differed in some cases, indicating the presence of heterogeneity in underlying cohort-specific estimates. For example, stunting incidence at ages peaked at ages 0-3 months in Latin America using random effects models, but in fixed effects models, incidence was similar at ages 0-3 and 3-6 months. However, overall, our scientific inferences from results produced by each method were similar.

6.1 Age-specific prevalence

6.1.1 Random effects

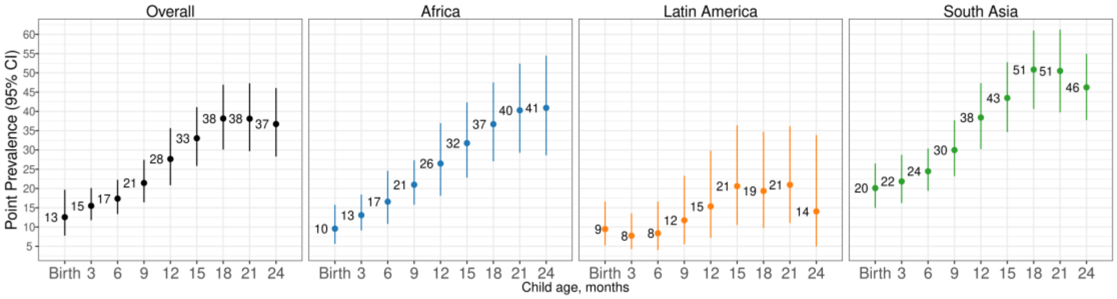

6.1.2 Fixed effects

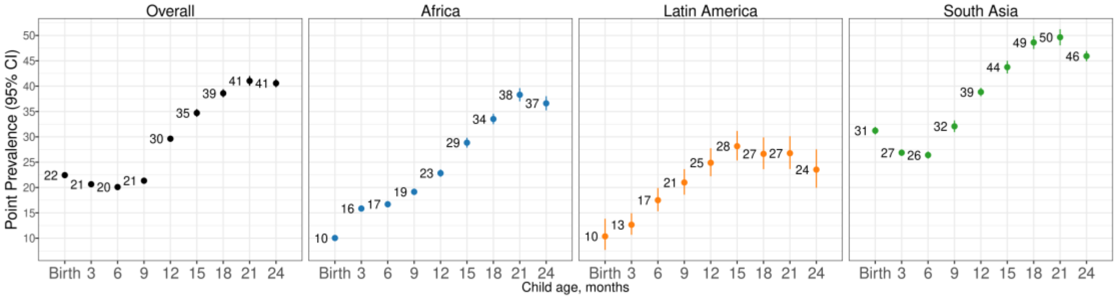

---

[1] Hedges, L. V. & Vevea, J. L. Fixed- and random-effects models in meta-analysis. Psychol. Methods 3, 486–504 (1998).

6.2 Age-specific incidence

6.2.1 Random effects

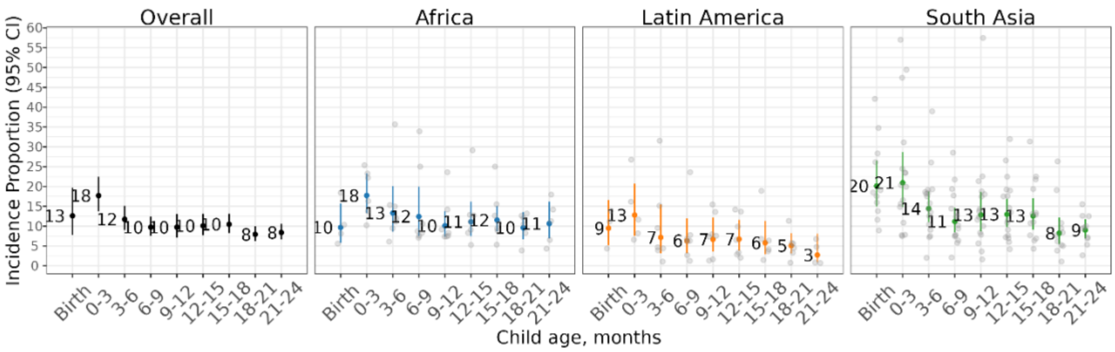

6.2.2 Fixed effects

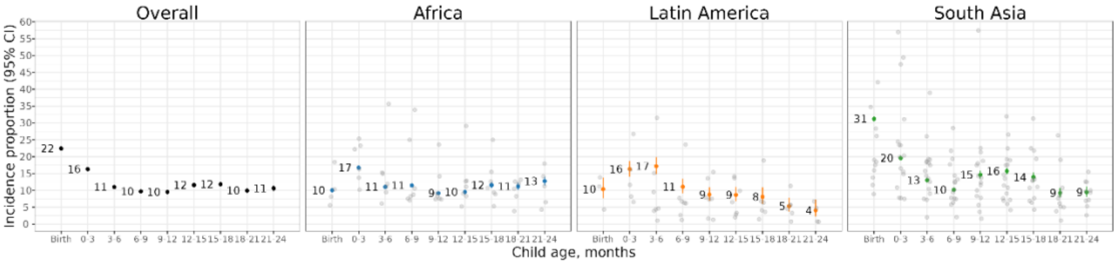

6.3 Changes in stunting status by age

6.3.1 Random effects

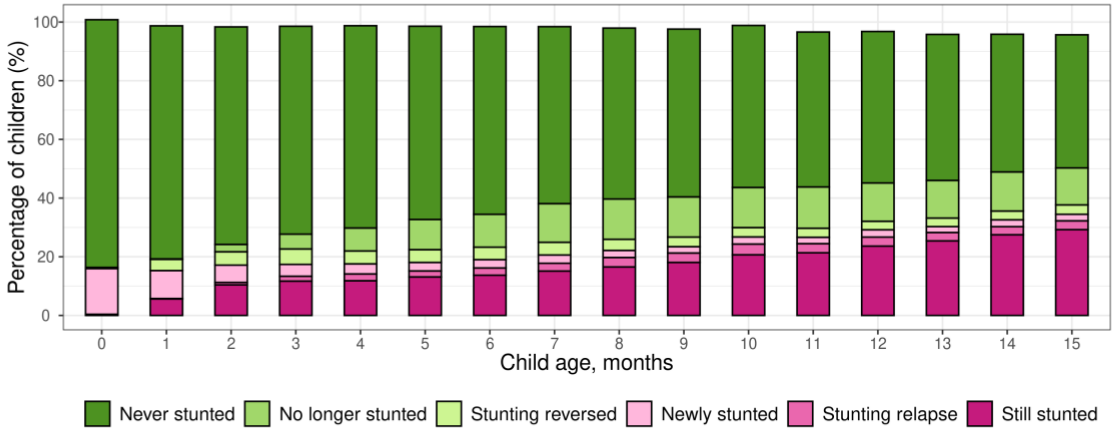

6.3.2 Fixed effects

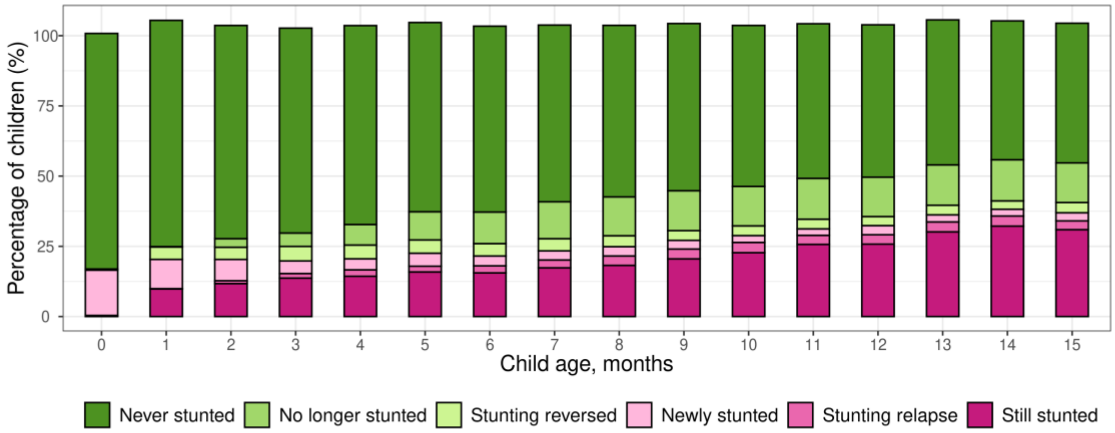

6.4 Linear growth velocity

6.4.1 Random effects

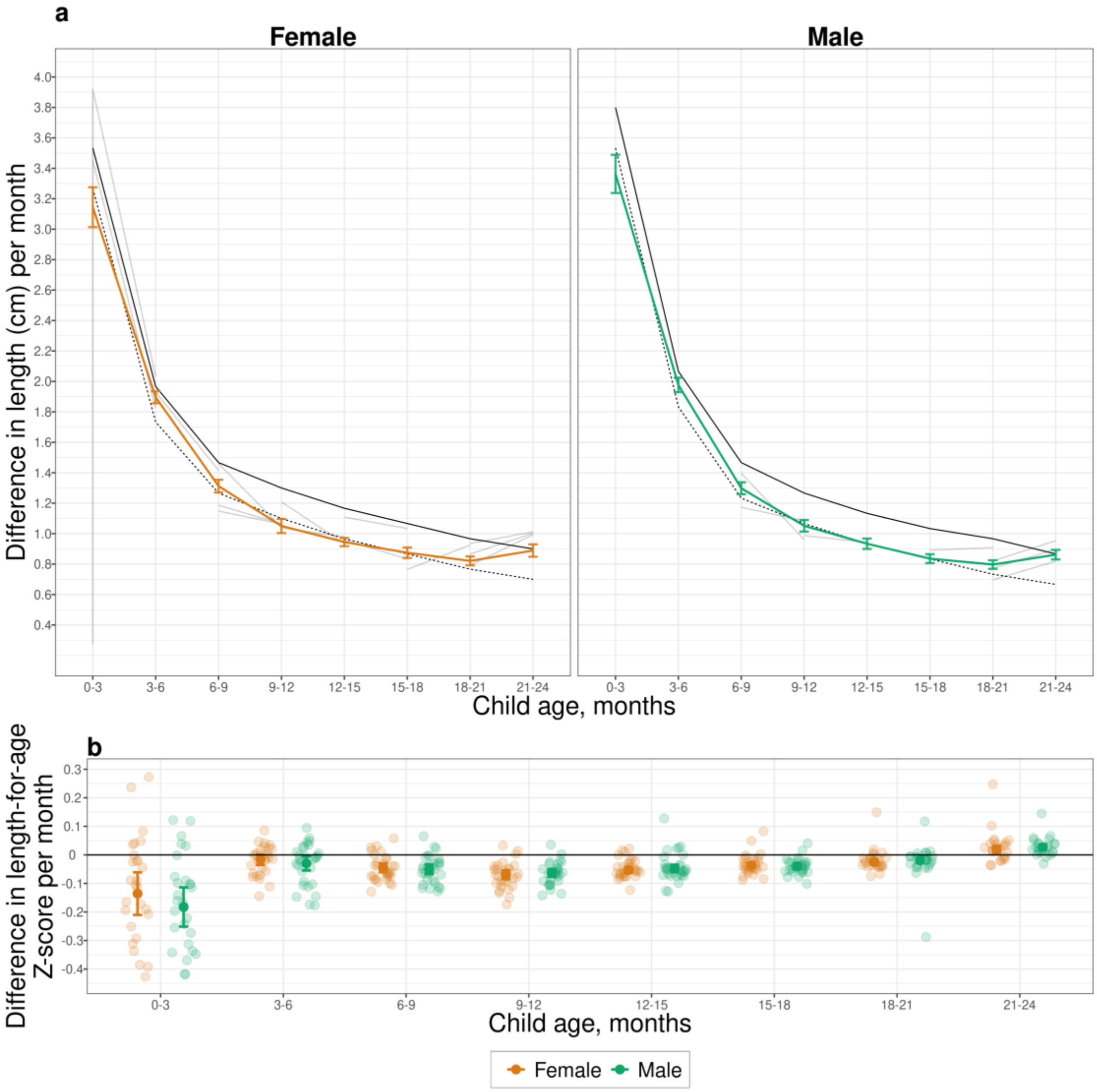

6.4.2 Fixed effects

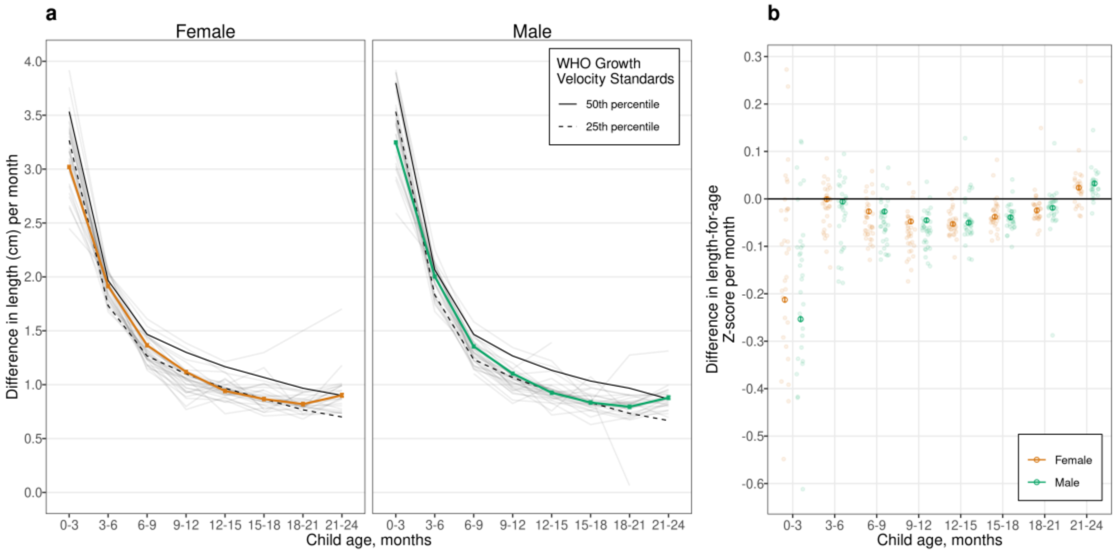

Supplementary Note 7. Cohort-specific results

Here, we present cohort-specific estimates of length-for-age Z-score by age, age-specific prevalence, and age-specific incidence.

7.1 Mean length-for-age Z-score by age

7.1.1 Africa

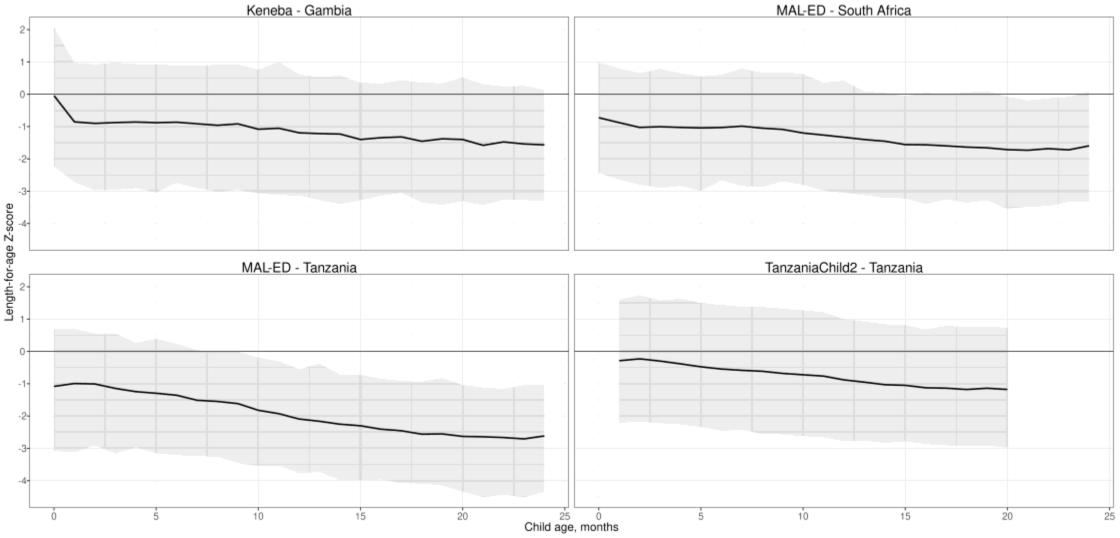

7.1.2 Latin America

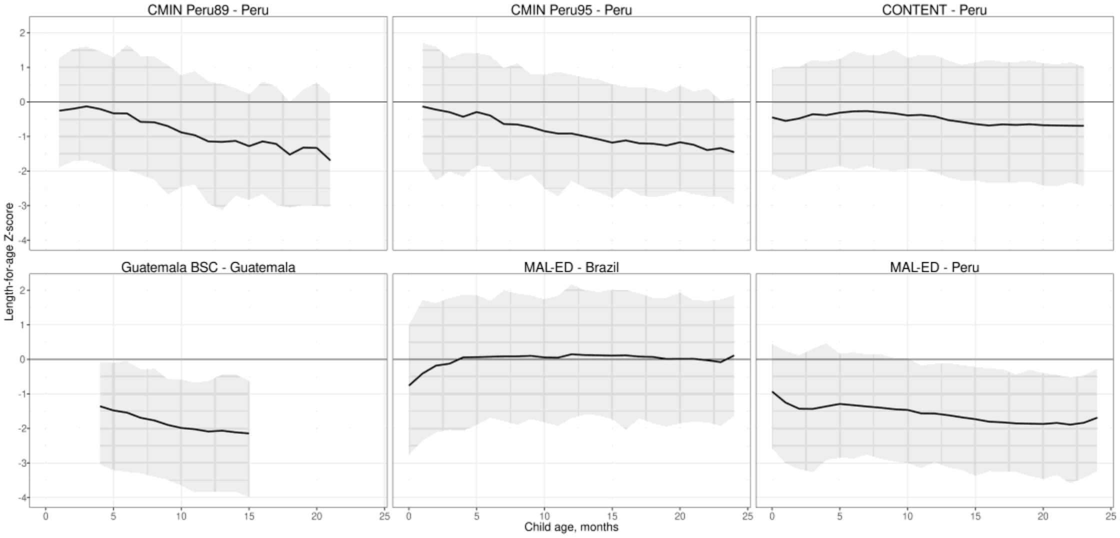

7.1.3 South Asia

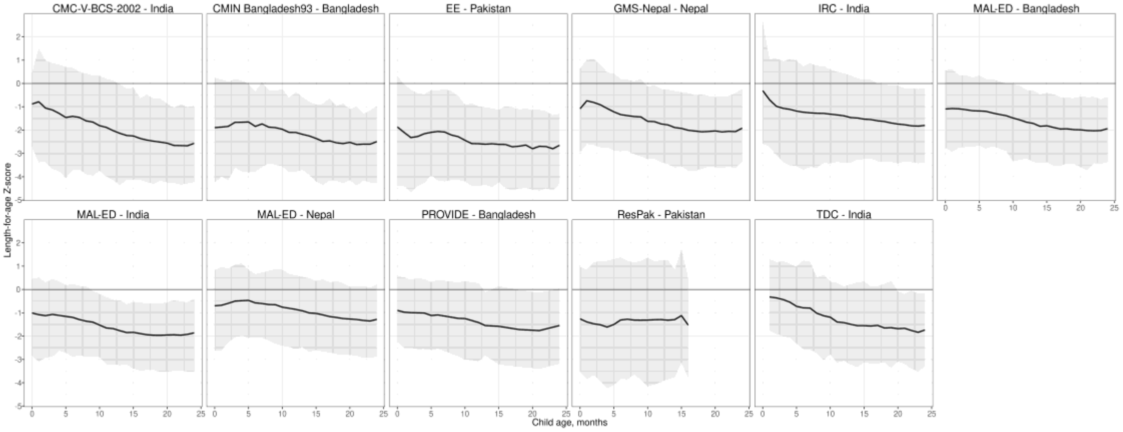

7.2 Age-specific prevalence

7.2.1 Africa

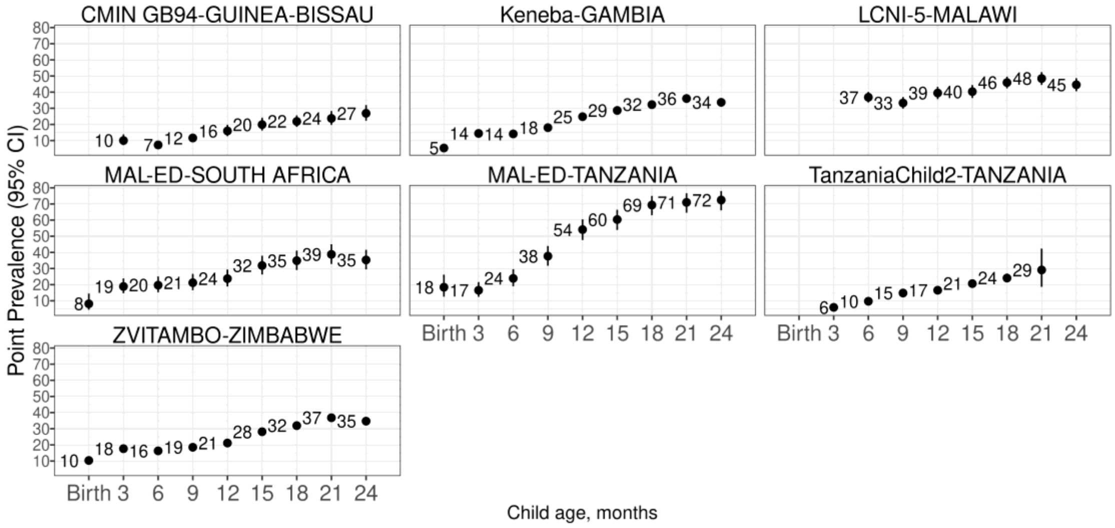

7.2.2 Latin America

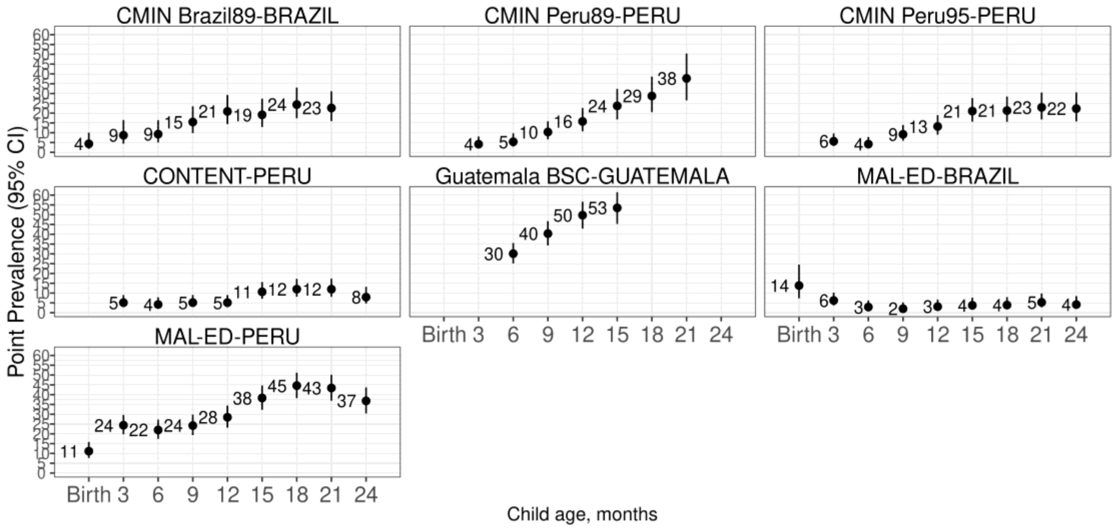

7.2.3 South Asia

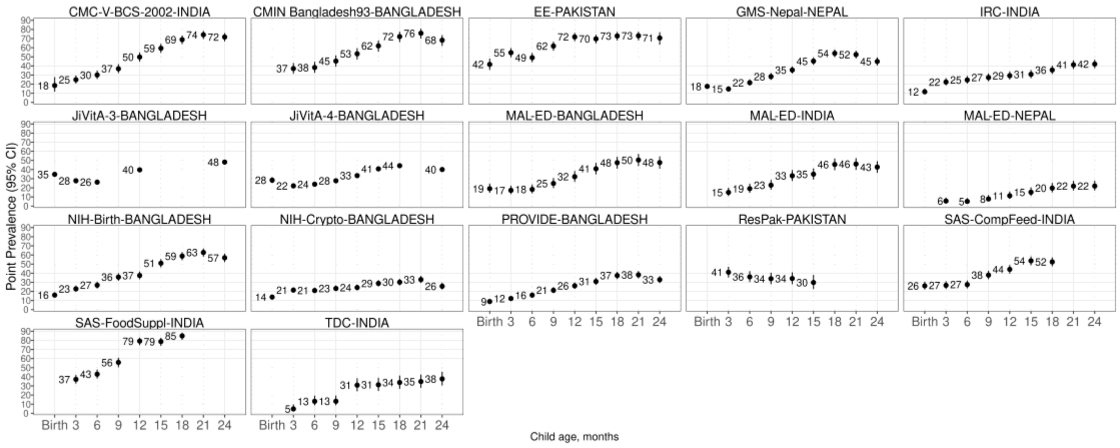

7.3 Age-specific incidence

7.3.1 Africa

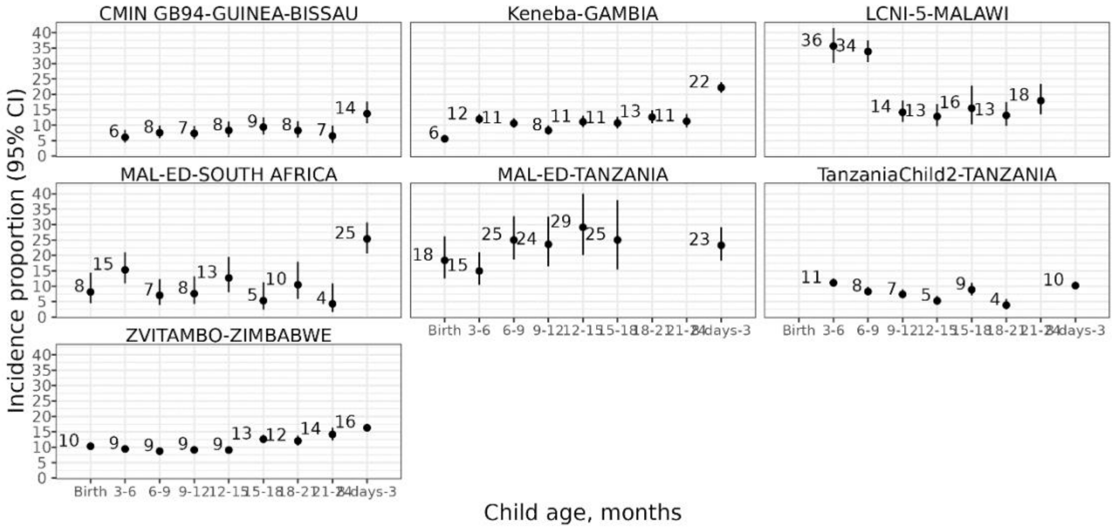

7.3.2 Latin America

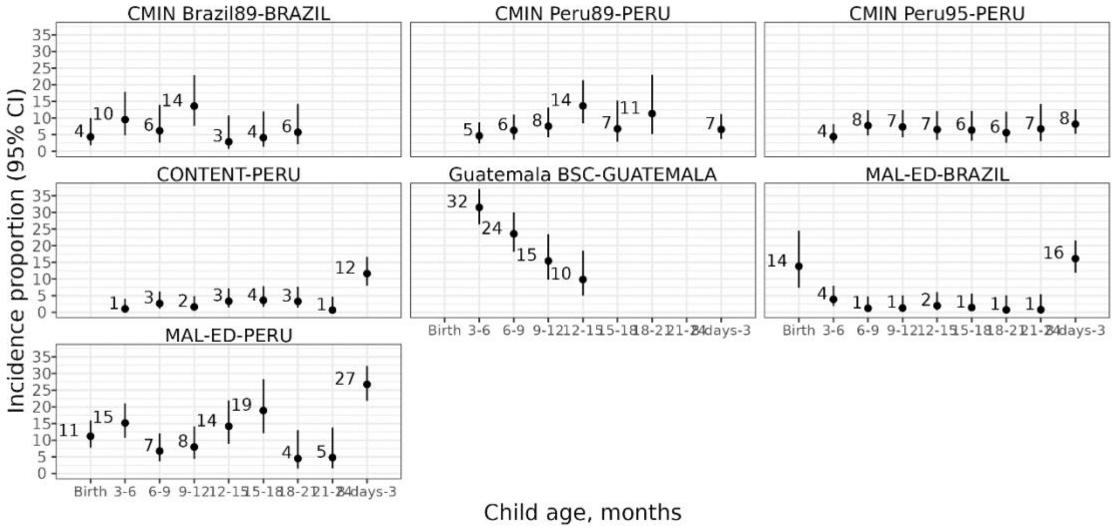

7.3.3 South Asia

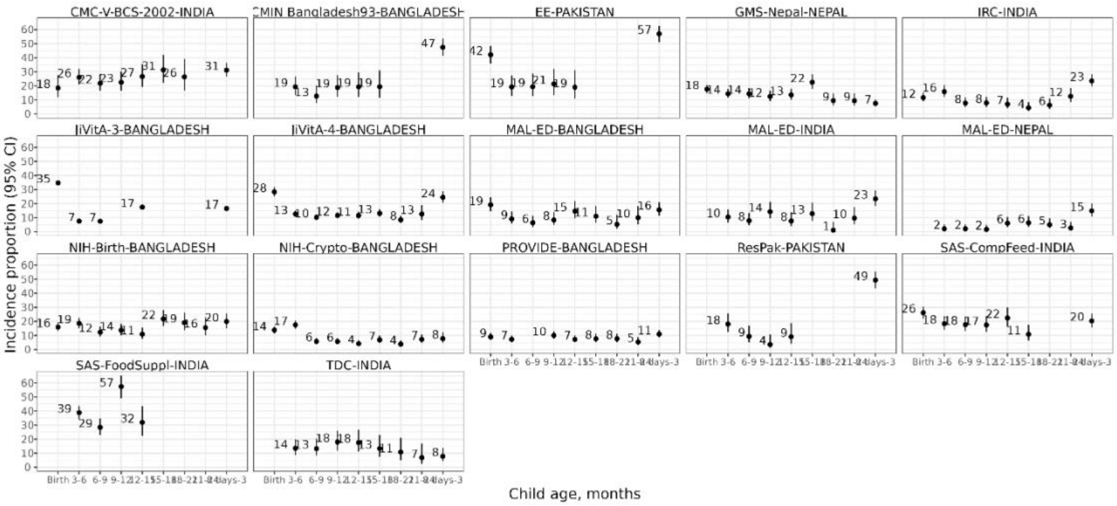

7.4 Length velocity by age and sex

7.4.1 Africa

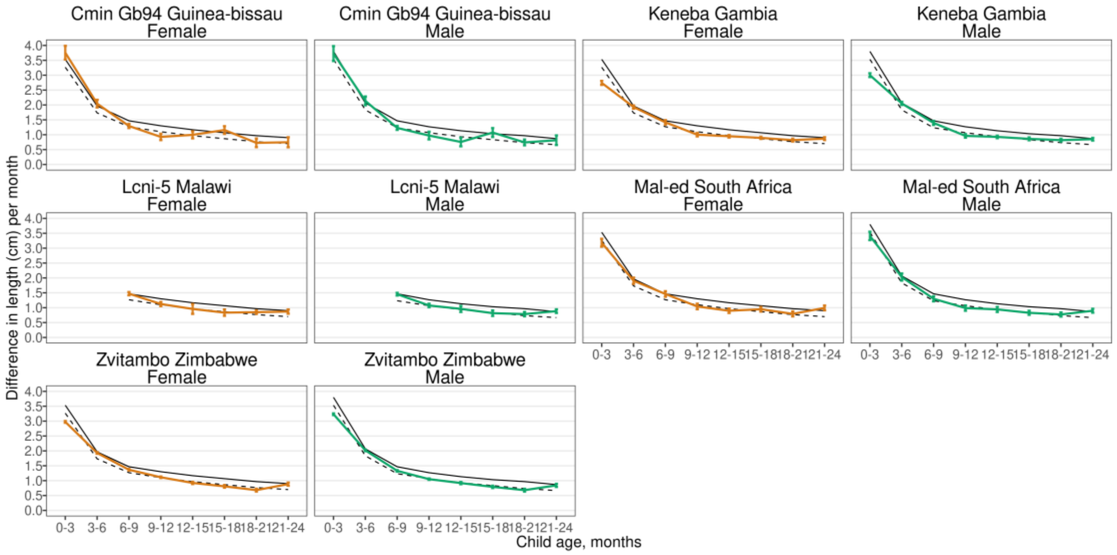

7.4.2 Latin America

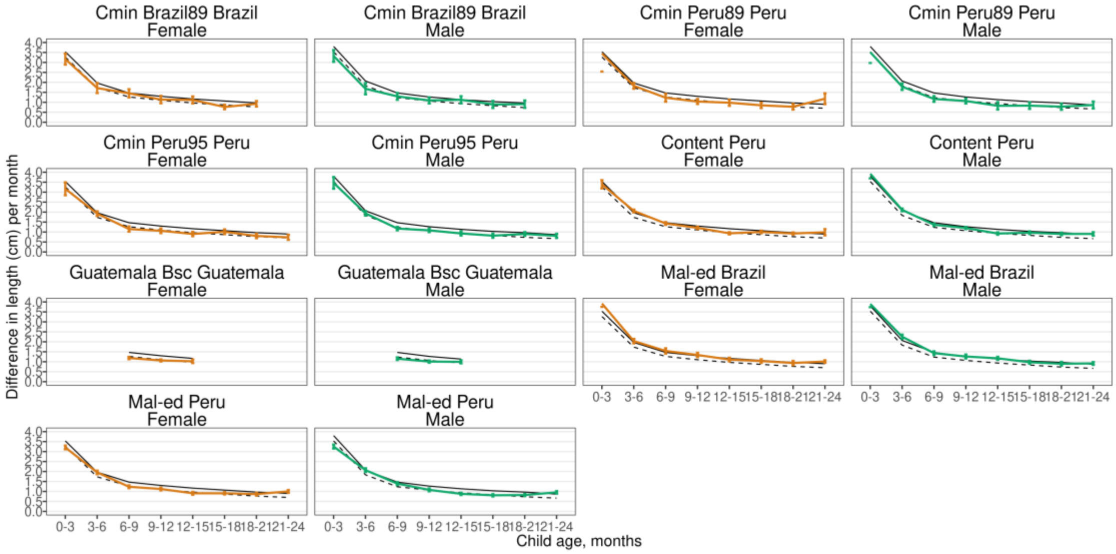

7.4.3 South Asia

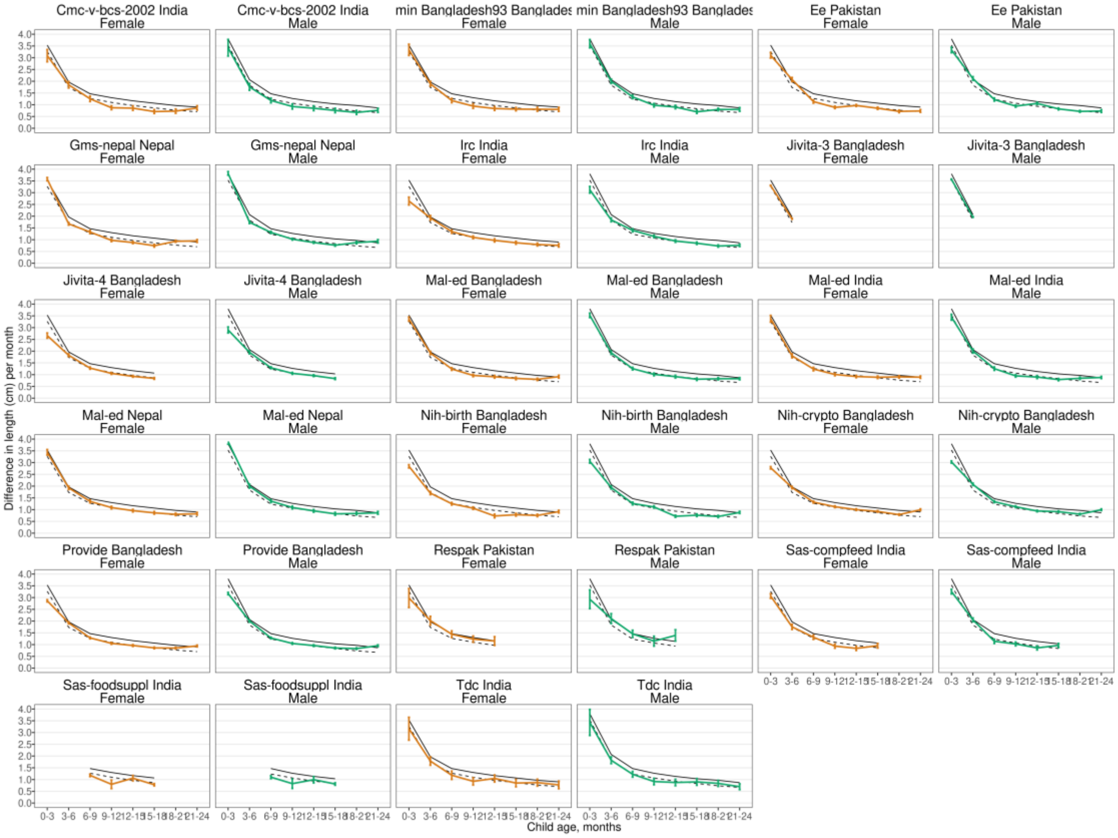

7.5 LAZ velocity by age and sex

7.5.1 Africa

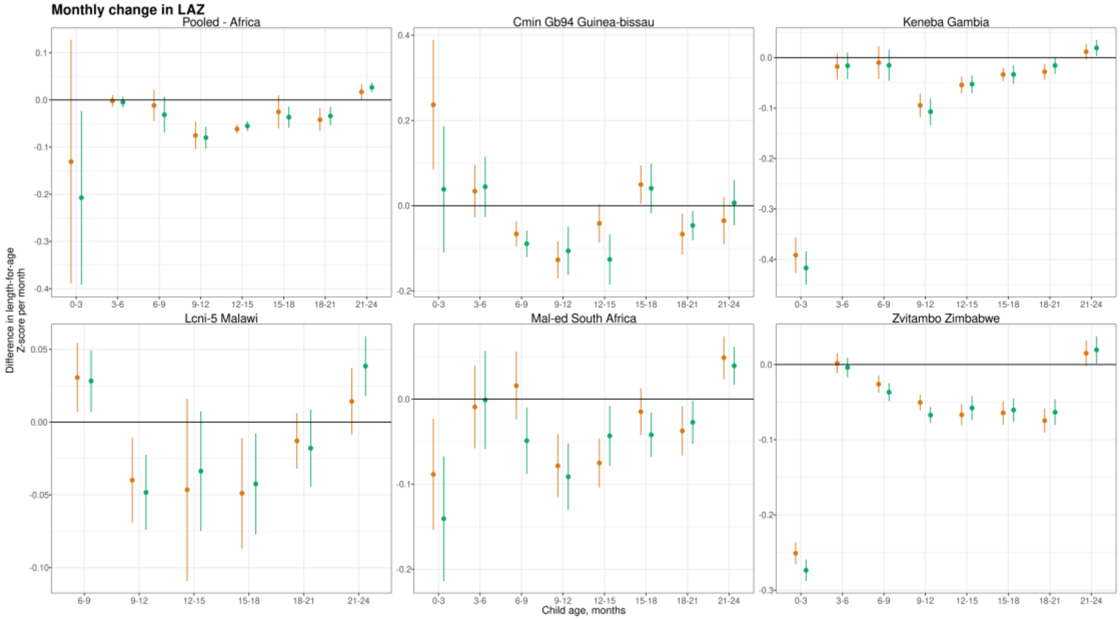

7.5.2 Latin America

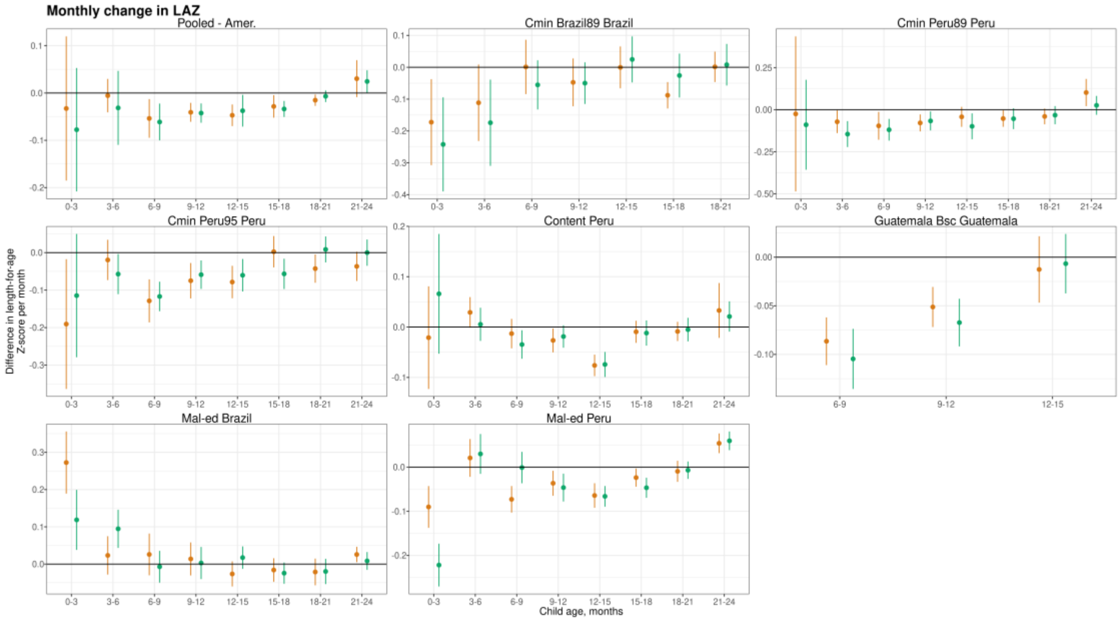

7.5.3 South Asia

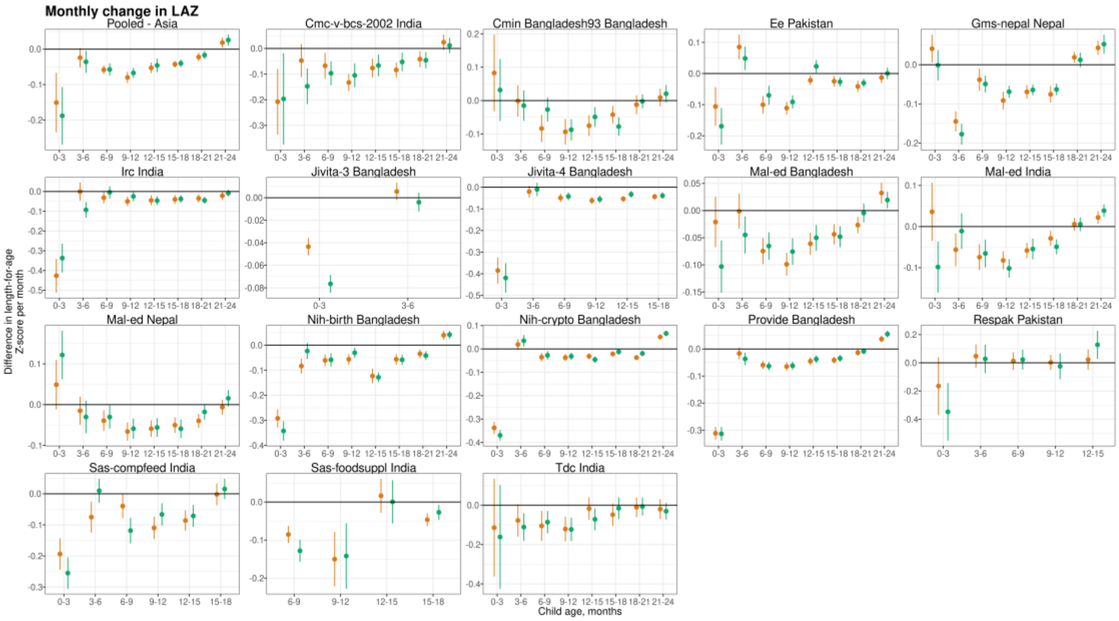

Supplementary Note 8. Primary analyses excluding the PROBIT study

Only one cohort from Europe met the inclusion criteria for this study – the PROBIT study. To assess whether inclusion of this study altered our overall study inference, we repeated analyses excluding the PROBIT cohort, as shown below in the "Overall" panels. Results were very similar with and without the PROBIT cohort. Stunting prevalence and incidence were slightly higher at birth when excluding PROBIT, but overall age-specific patterns remained the same. For this reason, we chose to retain PROBIT in the primary analyses presented in this manuscript.

8.1 Mean length-for-age Z-score by age

8.1.1 Including PROBIT

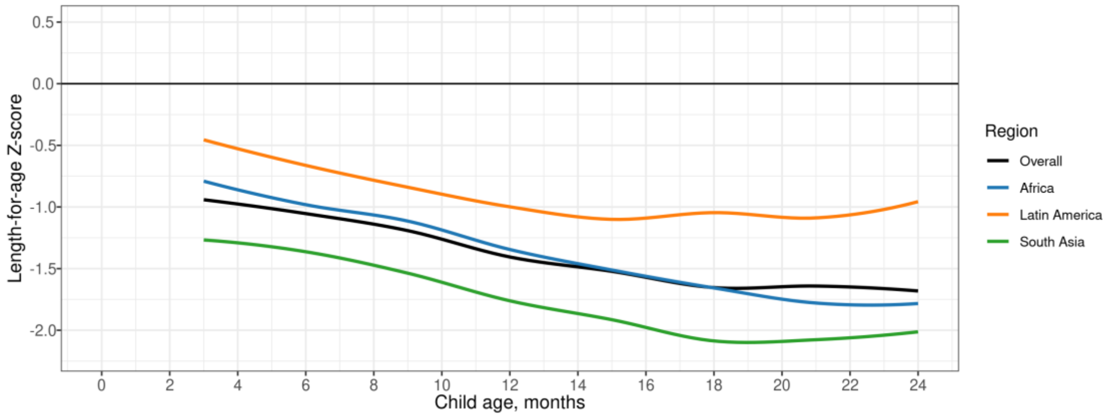

8.1.2 Excluding PROBIT

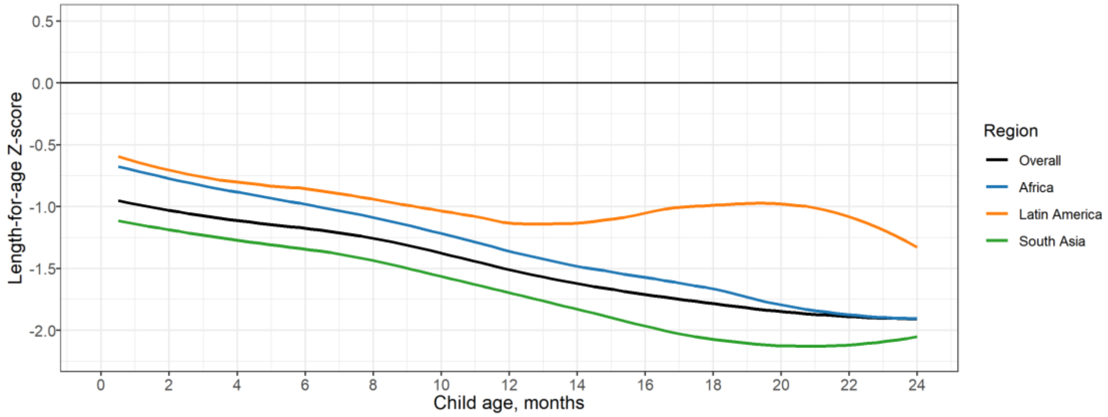

8.2 Age-specific prevalence

8.2.1 Including PROBIT

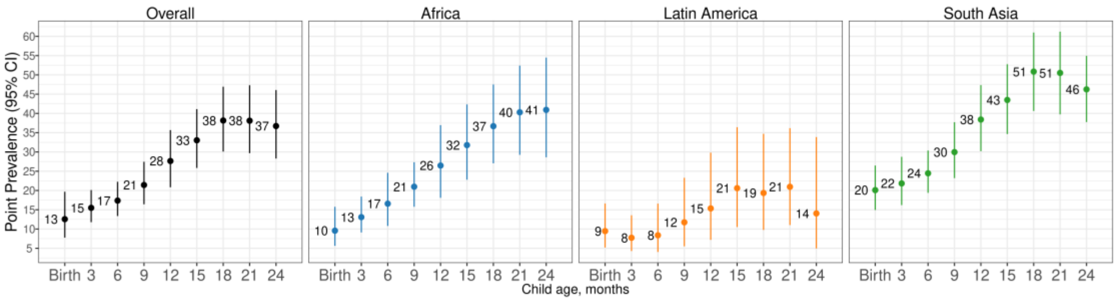

8.2.2 Excluding PROBIT

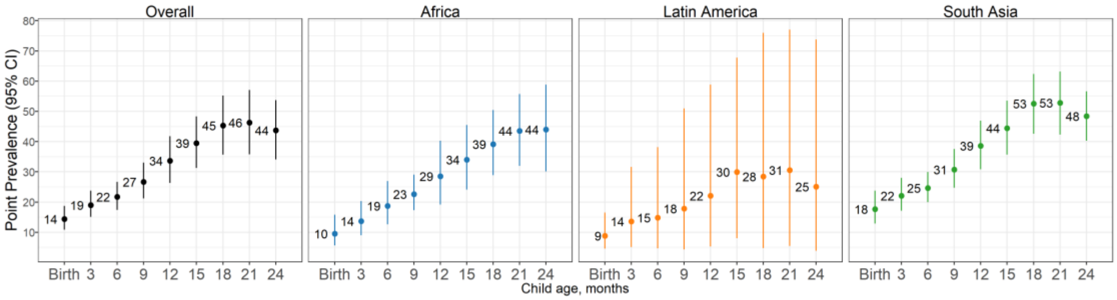

8.3 Age-specific incidence

8.3.1 Including PROBIT

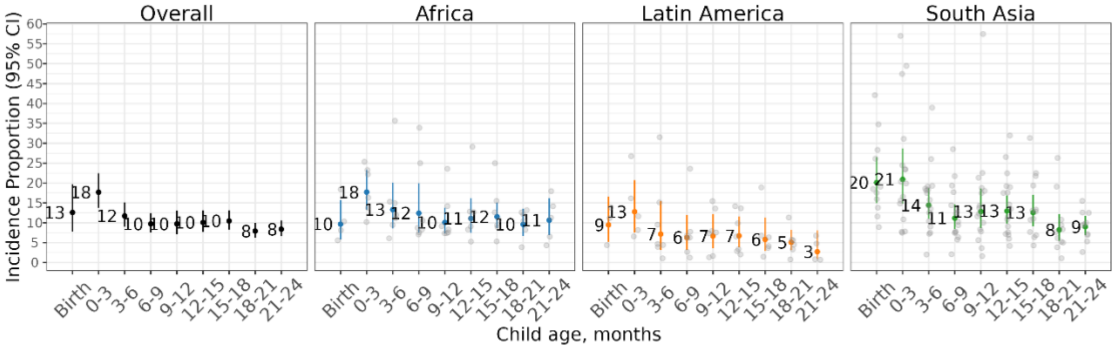

8.3.2 Excluding PROBIT

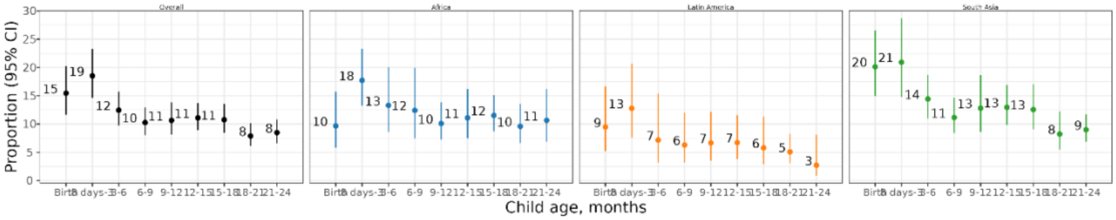

Supplementary Table 1. Summary of *ki* cohorts

Region, Study ID	Country	Study Years	Design	Children Enrolled*	Anthropometry measurement ages (months)	Total measurements*	Primary References
**South Asia**							
Biomarkers for EE	Pakistan	2013-2015	Prospective cohort	379	Birth, 1, 2, ..., 18	8484	Iqbal et al 2018 Nature Scientific Reports[1]
Resp. Pathogens	Pakistan	2011 - 2014	Prospective cohort	284	Birth, 1, 2, ..., 17	3177	Ali et al 2016 Journal of Medical Virology[2]
Growth Monitoring Study	Nepal	2012 - Ongoing	Prospective cohort	698	Birth, 1, 2, ..., 24	13465	Not yet published
MAL-ED	Nepal	2010 - 2014	Prospective cohort	240	Birth, 1, 2, ..., 24	5703	Shrestha et al 2014 Clin Infect Dis[3]
CMC Birth Cohort, Vellore	India	2002 - 2006	Prospective cohort	373	Birth, 0.5, 1, 1.5, ..., 24	8709	Gladstone et al. 2011 NEJM[4]
MAL-ED	India	2010 - 2012	Prospective cohort	251	Birth, 1, 2, ..., 24	5702	John et al 2014 Clin Infect Dis[5]
Vellore Crypto Study	India	2008 - 2011	Prospective cohort	410	Birth, 1, 2, ..., 24	9771	Kattula et al. 2014 BMJ Open[6]
CMIN 93	Bangladesh	1993 - 1996	Prospective Cohort	277	Birth, 3, 6, ..., 24	5363	Pathela et al 2007 Acta Paediatrica[7]
TDC	India	2008-2011	Quasi-experimental	160	Birth, 1, 2, ..., 24	3593	Sarkar et al. 2013 BMC Public Health [8]

MAL-ED	Bangladesh	2010 - 2014	Prospective cohort	265	Birth, 1, 2, ..., 24	5604	Ahmed et al 2014 Clin Infect Dis[9]
PROVIDE RCT	Bangladesh	2011 - 2014	Individual RCT	700	Birth, 6, 10, 12, 14. 17, 18, 24, 39, 40, 52, 53 (weeks)	9207	Kirkpatrick et al 2015 Am J Trop Med Hyg[10]
Food Suppl RCT	India	1995 - 1996	Individual RCT	418	Baseline, 6, 9, 12	2232	Bhandari et al 2001 J Nutri[11]
Optimal Infant Feeding	India	1999 - 2001	Cluster RCT	472	Birth, 3, 6, ..., 18	2948	Bhandari et al 2004 J Nutri[12]
NIH Birth Cohort	Bangladesh	2008 - 2009	Prospective Cohort	629	Birth, 3, 6, ..., 12	6215	Korpe et al. 2016 PLOS NTD[13]
JiVitA-4 Trial	Bangladesh	2012 - 2014	Cluster RCT	1434	6, 9, 12, 14, 18	9344	Christian et al 2015 IJE[14]
JiVitA-3 Trial	Bangladesh	2008 - 2012	Cluster RCT	13475	Birth, 1, 3, 6, 12, 24	53453	West et al JAMA 2014[15]
NIH Cryptosporidium Study	Bangladesh	2014 - 2017	Prospective cohort	758	Birth, 3, 6, ..., 24	6914	Steiner et al 2018 Clin Infect Dis[16]
**Africa**							
MAL-ED	Tanzania	2009 - 2014	Prospective cohort	262	Birth, 1, 2, ..., 24	5713	Mduma et al 2014 Clin Infect Dis[17]
Tanzania Child 2	Tanzania	2007 - 2011	Individual RCT	2396	1, 2, ..., 20	29565	Locks et al Am J Clin Nutr 2016[18]

MAL-ED	South Africa	2009 - 2014	Prospective cohort	314	Birth, 1, 2, ..., 24	6163	Bessong et al 2014 Clin Infect Dis[19]
MRC Keneba	Gambia	1987 - 1997	Cohort	2915	Birth, 1, 2, ..., 24	40275	Schoenbuchner et al. 2019, AJCN[20]
ZVITAMBO Trial	Zimbabwe	1997 - 2001	Individual RCT	14074	Birth, 6 wks, 3, 6, 9, 12	71928	Malaba et al 2005 Am J Clin Nutr[21]
Lungwena Child Nutrition RCT	Malawi	2011 - 2014	Individual RCT	840	Birth, 1-6 wk, 6, 12 18	4336	Mangani et al. 2015, Mat Child Nutr[22]
iLiNS-Zinc Study	Burkina Faso	2010 - 2012	Cluster RCT	797	9, 12, 15, 18	1471	Hess et al 2015 Plos One[23]
CMIN GB94	Guinea Bissau	1994 - 1997	Prospective Cohort	870	Enrollment and every 3 months after	6451	Valentiner-Branth 2001 Am J Clin Nutr[24]
**Latin America**							
MAL-ED	Peru	2009 - 2014	Prospective cohort	303	Birth, 1, 2, ..., 24	6142	Yori et al 2014 Clin Infect Dis[25]
CONTENT	Peru	2007 - 2011	Prospective cohort	215	Birth, 1, 2, ..., 24	8339	Jaganath et al 2014 Helicobacter[26]
Bovine Serum RCT	Guatemala	1997 - 1998	Individual RCT	315	Baseline, 1, 2, ...,8	2545	Begin et al. 2008, EJCN[27]
MAL-ED	Brazil	2010 - 2014	Prospective cohort	233	Birth, 1, 2, ..., 24	4858	Lima et al 2014 Clin Infect Dis[28]
CMIN Brazil89	Brazil	1989-2000	Prospective Cohort	119	Birth, 1, 2, ..., 24	889	Moore et al. 2001 Int J Epidemiol.[29]

CMIN Peru95	Peru	1995 - 1998	Prospective Cohort	224	Birth, 1, 2, ..., 24	3979	Checkley et al. 2003 Am J Epidemiol.[30]
CMIN Peru89	Peru	1989 - 1991	Prospective Cohort	210	Birth, 1, 2, ..., 24	2742	Checkley et al. 1998 Am J Epidemiol.[31]
**Europe**							
PROBIT Study	Belarus	1996 - 1997	Cluster RCT	8127	1, 2, 3, 6, 9, 12	58649	Kramer et al 2001 JAMA[32]
*Children enrolled is for children with measurements under 2 years of age. Total measurements are number of measurements of anthropometry on children under 2 years of age.							

Supplementary Table 2. Characteristics of participants in each cohort

	CMC-V-BCS-2002, India	CMIN Bangladesh 93	CMIN Brazil 89	CMIN Guinea-Bissau 94	CMIN Peru 89	CMIN Peru 95	CONTENT, Peru	EE, Pakistan	GMS-Nepal	Guatemala BSC
	(N=373)	(N=280)	(N=119)	(N=885)	(N=210)	(N=224)	(N=215)	(N=380)	(N=698)	(N=315)
Sex										
Female	187 (50.1%)	122 (43.6%)	62 (52.1%)	443 (50.1%)	100 (47.6%)	96 (42.9%)	109 (50.7%)	185 (48.7%)	328 (47.0%)	162 (51.4%)
Male	186 (49.9%)	158 (56.4%)	57 (47.9%)	442 (49.9%)	110 (52.4%)	128 (57.1%)	106 (49.3%)	195 (51.3%)	370 (53.0%)	153 (48.6%)
Birthweight										
Mean (SD)	2910 (434)	2560 (967)	3270 (477)	3330 (541)	3550 (492)	3690 (367)	3070 (323)	2640 (506)	2660 (423)	
Maternal age										
Mean (SD)	24.1 (4.11)							30.0 (3.99)	24.0 (5.09)	25.2 (6.21)
Maternal weight										
Mean (SD)										
Maternal education (years)										
Mean (SD)	5.43 (4.10)							0.873 (2.51)	2.48 (4.01)	4.07 (2.95)
Number of rooms										
4+	14 (3.75%)						78 (36.3%)		323 (46.3%)	
1	202 (54.2%)						44 (20.5%)		49 (7.02%)	
2	106 (28.4%)						54 (25.1%)		145 (20.8%)	
3	51 (13.7%)						39 (18.1%)		181 (25.9%)	
Number of children <5yrs										
1										
2+										
Improved sanitation										
1							201 (93.5%)			
0							14 (6.51%)			
Food security level										
Food Secure									479 (71.1%)	
Mildly Food Insecure									106 (15.7%)	
Food Insecure									89 (13.2%)	

	IRC, India	JiVitA-3, Bangladesh	JiVitA-4, Bangladesh	Keneba, Gambia	LCNI-5, Malawi	MAL-ED, Bangladesh	MAL-ED, Brazil	MAL-ED, India	MAL-ED, Nepal	MAL-ED, Peru	MAL-ED, South Africa
	(N=410)	(N=28300)	(N=5449)	(N=2954)	(N=840)	(N=265)	(N=233)	(N=251)	(N=240)	(N=303)	(N=314)
Sex											
Female	185 (45.1%)	13785 (48.7%)	2728 (50.1%)	1426 (48.3%)	421 (50.1%)	136 (51.3%)	113 (48.5%)	138 (55.0%)	110 (45.8%)	143 (47.2%)	159 (50.6%)
Male	225 (54.9%)	14515 (51.3%)	2721 (49.9%)	1528 (51.7%)	419 (49.9%)	129 (48.7%)	120 (51.5%)	113 (45.0%)	130 (54.2%)	160 (52.8%)	155 (49.4%)
Birthweight											
Mean (SD)	2890 (448)	2550 (428)	2990 (823)	2970 (421)		2800 (412)	3340 (483)	2890 (442)	2980 (390)	3130 (430)	3130 (464)
Maternal age											
Mean (SD)	23.7 (3.68)	21.4 (5.27)		27.4 (7.16)	26.8 (7.87)	24.8 (4.99)	24.8 (5.53)	23.9 (4.10)	26.4 (3.76)	24.2 (6.06)	26.4 (6.86)
Maternal weight											
Mean (SD)		43.7 (6.13)			50.9 (6.66)	49.6 (8.54)	61.9 (11.8)	50.4 (9.37)	56.3 (8.27)	55.5 (8.95)	67.5 (14.9)
Maternal education (years)											
Mean (SD)	5.06 (4.61)	5.06 (3.82)	7.51 (4.82)		3.54 (3.34)	5.63 (2.58)	9.18 (2.81)	7.88 (3.17)	8.77 (3.44)	7.81 (2.79)	10.3 (1.94)
Number of rooms											
4+	17 (4.17%)	1181 (4.18%)	286 (5.26%)			12 (4.96%)	127 (60.5%)	25 (10.6%)	131 (55.5%)	139 (51.1%)	196 (76.3%)
1	185 (45.3%)	16754 (59.3%)	3196 (58.8%)			152 (62.8%)	4 (1.90%)	84 (35.7%)	52 (22.0%)	19 (6.99%)	14 (5.45%)
2	170 (41.7%)	7605 (26.9%)	1389 (25.6%)			50 (20.7%)	20 (9.52%)	78 (33.2%)	31 (13.1%)	52 (19.1%)	22 (8.56%)
3	36 (8.82%)	2723 (9.63%)	562 (10.3%)			28 (11.6%)	59 (28.1%)	48 (20.4%)	22 (9.32%)	62 (22.8%)	25 (9.73%)
Number of children <5yrs											
1	89 (21.7%)	18200 (64.4%)			391 (48.2%)						
2+	321 (78.3%)	10066 (35.6%)			421 (51.8%)						
Improved sanitation											
1		19609 (69.4%)	4191 (77.1%)		3 (0.369%)	204 (84.3%)	206 (98.1%)	108 (46.4%)	235 (99.6%)	65 (24.7%)	4 (1.60%)
0		8655 (30.6%)	1242 (22.9%)		810 (99.6%)	38 (15.7%)	4 (1.90%)	125 (53.6%)	1 (0.424%)	198 (75.3%)	246 (98.4%)
Food security level											
Food Secure		8711 (50.0%)	2751 (50.9%)			161 (83.0%)	3 (2.33%)	190 (89.6%)	94 (73.4%)	27 (23.9%)	132 (56.7%)
Mildly Food Insecure		5793 (33.3%)	1951 (36.1%)			4 (2.06%)	11 (8.53%)	5 (2.36%)	15 (11.7%)	29 (25.7%)	19 (8.15%)
Food Insecure		2910 (16.7%)	703 (13.0%)			29 (14.9%)	115 (89.1%)	17 (8.02%)	19 (14.8%)	57 (50.4%)	82 (35.2%)

	MAL-ED, Tanzania	NIH-Birth, Bangladesh	NIH-Crypto, Bangladesh	PROBIT, Belarus	PROVIDE, Bangladesh	ResPak, Pakistan	SAS-CompFeed, India	SAS-FoodSuppl, India	Tanzania Child 2	TDC, India	ZVITAMBO, Zimbabwe
	(N=262)	(N=629)	(N=758)	(N=17046)	(N=700)	(N=284)	(N=1535)	(N=418)	(N=2400)	(N=160)	(N=14104)
Sex											
Female	133 (50.8%)	297 (47.2%)	381 (50.3%)	8217 (48.2%)	332 (47.4%)	136 (47.9%)	702 (45.7%)	223 (53.3%)	1184 (49.3%)	75 (46.9%)	6847 (48.5%)
Male	129 (49.2%)	332 (52.8%)	377 (49.7%)	8829 (51.8%)	368 (52.6%)	148 (52.1%)	833 (54.3%)	195 (46.7%)	1216 (50.7%)	85 (53.1%)	7257 (51.5%)
Birthweight											
Mean (SD)	3180 (452)	2710 (414)	2800 (410)	3440 (419)	2780 (371)	2930 (525)	2750 (516)		3230 (474)	2910 (450)	2970 (456)
Maternal age											
Mean (SD)	28.4 (6.67)	25.0 (5.32)	24.3 (4.68)	24.9 (4.92)	24.7 (4.65)		23.6 (4.08)	25.0 (4.63)	26.4 (5.04)		24.5 (5.30)
Maternal weight											
Mean (SD)	55.7 (9.11)	48.0 (8.51)	52.1 (9.17)	66.2 (12.5)	49.3 (9.41)		47.6 (7.05)		62.2 (11.7)		60.0 (11.1)
Maternal education (years)											
Mean (SD)	6.17 (1.79)	3.76 (3.45)	5.77 (3.63)	13.0 (1.40)	4.33 (3.62)		4.42 (4.52)	1.25 (2.70)	7.90 (2.27)		9.66 (1.95)
Number of rooms											
4+	108 (43.2%)		95 (12.5%)		23 (3.29%)					5 (3.13%)	
1	13 (5.20%)		368 (48.5%)		507 (72.4%)					91 (56.9%)	
2	63 (25.2%)		191 (25.2%)		108 (15.4%)					49 (30.6%)	
3	66 (26.4%)		104 (13.7%)		62 (8.86%)					15 (9.38%)	
Number of children <5yrs											
1			541 (71.4%)		512 (73.1%)				1640 (68.6%)		
2+			217 (28.6%)		188 (26.9%)				749 (31.4%)		
Improved sanitation											
1		242 (38.5%)	655 (87.7%)		58 (96.7%)						
0		387 (61.5%)	92 (12.3%)		2 (3.33%)						
Food security level											
Food Secure		95 (15.1%)	453 (59.8%)								
Mildly Food Insecure		492 (78.2%)	219 (28.9%)								
Food Insecure		42 (6.68%)	86 (11.3%)								

Supplementary Table 3. Number of children included by subgroup and region

	Africa (N=21759)	South Asia (N=40279)	Latin America (N=1619)	Europe (N=16898)	Overall (N=80555)
**Decade**					
1990s	16364 (75.2%)	2233 (5.5%)	868 (53.6%)	16898 (100%)	36363 (45.1%)
2000s	5063 (23.3%)	23108 (57.4%)	423 (26.1%)	0 (0%)	28594 (35.5%)
2010s	332 (1.5%)	14938 (37.1%)	328 (20.3%)	0 (0%)	15598 (19.4%)
**Gross domestic product per capita (in millions of USD)**					
< $1,026	21445 (98.6%)	38119 (94.6%)	0 (0%)	0 (0%)	59564 (73.9%)
≥ $1,026	314 (1.4%)	2160 (5.4%)	1619 (100%)	16898 (100%)	20991 (26.1%)
**Gender development index**					
< 10	2223 (10.2%)	25265 (62.7%)	0 (0%)	0 (0%)	27488 (34.1%)
10 – 16	14898 (68.5%)	15014 (37.3%)	315 (19.5%)	0 (0%)	30227 (37.5%)
≥ 16	821 (3.8%)	0 (0%)	1009 (62.3%)	16898 (100%)	18728 (23.2%)
Missing	3817 (17.5%)	0 (0%)	295 (18.2%)	0 (0%)	4112 (5.1%)
**Gender inequality index**					
< 0.596	10378 (47.7%)	2606 (6.5%)	0 (0%)	0 (0%)	12984 (16.1%)
0.596 – 0.614	7250 (33.3%)	14604 (36.3%)	0 (0%)	0 (0%)	21854 (27.1%)
≥ 0.614	314 (1.4%)	23069 (57.3%)	1522 (94.0%)	0 (0%)	24905 (30.9%)
Missing	3817 (17.5%)	0 (0%)	97 (6.0%)	16898 (100%)	20812 (25.8%)
**Coefficient of human inequality**					
< 28.3	2117 (9.7%)	7255 (18.0%)	358 (22.1%)	0 (0%)	9730 (12.1%)
28.3 – 29.3	0 (0%)	9959 (24.7%)	0 (0%)	0 (0%)	9959 (12.4%)
≥ 29.3	0 (0%)	20459 (50.8%)	393 (24.3%)	0 (0%)	20852 (25.9%)
Missing	19642 (90.3%)	2606 (6.5%)	868 (53.6%)	16898 (100%)	40014 (49.7%)
**GINI coefficient**					
< 32.32	0 (0%)	30713 (76.3%)	0 (0%)	0 (0%)	30713 (38.1%)
32.32 – 32.54	0 (0%)	5899 (14.6%)	0 (0%)	16898 (100%)	22797 (28.3%)
≥ 32.54	7107 (32.7%)	3667 (9.1%)	1409 (87.0%)	0 (0%)	12183 (15.1%)
Missing	14652 (67.3%)	0 (0%)	210 (13.0%)	0 (0%)	14862 (18.4%)
**Total expenditure on health (as % of gross domestic product)**					
1 – 3%	0 (0%)	28901 (71.8%)	0 (0%)	0 (0%)	28901 (35.9%)
3 – 5%	1337 (6.1%)	11098 (27.6%)	742 (45.8%)	0 (0%)	13177 (16.4%)
≥ 5%	5399 (24.8%)	0 (0%)	582 (35.9%)	16898 (100%)	22879 (28.4%)
Missing	15023 (69.0%)	280 (0.7%)	295 (18.2%)	0 (0%)	15598 (19.4%)
**% of population living on below $1.90 per day**					
< 18%	314 (1.4%)	7891 (19.6%)	1207 (74.6%)	16898 (100%)	26310 (32.7%)
18 – 28%	129 (0.6%)	29339 (72.8%)	202 (12.5%)	0 (0%)	29670 (36.8%)
≥ 28%	6664 (30.6%)	3049 (7.6%)	0 (0%)	0 (0%)	9713 (12.1%)
Missing	14652 (67.3%)	0 (0%)	210 (13.0%)	0 (0%)	14862 (18.4%)
**Under-5 mortality rate**					
<50 per 100,000	196 (0.9%)	22027 (54.7%)	852 (52.6%)	16898 (100%)	39973 (49.6%)
50-95 per 100,000	4250 (19.5%)	16591 (41.2%)	767 (47.4%)	0 (0%)	21608 (26.8%)
>95 per 100,000	17313 (79.6%)	1661 (4.1%)	0 (0%)	0 (0%)	18974 (23.6%)
