# Supplementary material for: Early-childhood linear growth faltering in low- and middle-income countries
Source: Nature. 2023 Sep 13;621(7979):550–7. doi: 10.1038/s41586-023-06418-5 (PMC10511325; doi:10.1038/s41586-023-06418-5)
Supplement: Supplementary file 2 — Reporting Summary [file 41586_2023_6418_MOESM2_ESM.pdf]

nature research

Corresponding author(s): Jade Benjamin-Chung

Last updated by author(s): 7/26/2024

Reporting Summary

Nature Research wishes to improve the reproducibility of the work that we publish. This form provides structure for consistency and transparency in reporting. For further information on Nature Research policies, see Authors & Referees and the Editorial Policy Checklist.

Statistics

For all statistical analyses, confirm that the following items are present in the figure legend, table legend, main text, or Methods section.

n/a	Confirmed	
☐	☒	The exact sample size (*n*) for each experimental group/condition, given as a discrete number and unit of measurement
☐	☒	A statement on whether measurements were taken from distinct samples or whether the same sample was measured repeatedly
☒	☐	The statistical test(s) used AND whether they are one- or two-sided<br>*Only common tests should be described solely by name; describe more complex techniques in the Methods section.*
☐	☒	A description of all covariates tested
☐	☒	A description of any assumptions or corrections, such as tests of normality and adjustment for multiple comparisons
☐	☒	A full description of the statistical parameters including central tendency (e.g. means) or other basic estimates (e.g. regression coefficient) AND variation (e.g. standard deviation) or associated estimates of uncertainty (e.g. confidence intervals)
☐	☒	For null hypothesis testing, the test statistic (e.g. *F*, *t*, *r*) with confidence intervals, effect sizes, degrees of freedom and *P* value noted<br>*Give P values as exact values whenever suitable.*
☒	☐	For Bayesian analysis, information on the choice of priors and Markov chain Monte Carlo settings
☐	☒	For hierarchical and complex designs, identification of the appropriate level for tests and full reporting of outcomes
☐	☒	Estimates of effect sizes (e.g. Cohen's *d*, Pearson's *r*), indicating how they were calculated

Our web collection on statistics for biologists contains articles on many of the points above.

Software and code

Policy information about availability of computer code

Data collection	This manuscript is a secondary data analysis of existing study data from 31 cohorts and trials, so we were not involved in original data collection.
Data analysis	All analyses were conducted using R statistical software, and scripts that reproduce all analyses are available on Github here: https://github.com/child-growth/ki-longitudinal-growth

For manuscripts utilizing custom algorithms or software that are central to the research but not yet described in published literature, software must be made available to editors/reviewers. We strongly encourage code deposition in a community repository (e.g. GitHub). See the Nature Research guidelines for submitting code & software for further information.

Data

Policy information about availability of data

All manuscripts must include a data availability statement. This statement should provide the following information, where applicable:

- Accession codes, unique identifiers, or web links for publicly available datasets
- A list of figures that have associated raw data
- A description of any restrictions on data availability

The data that support the findings of this study are available from the original study data contributors upon reasonable request (email contact: amertens@berkeley.edu).

Field-specific reporting

Please select the one below that is the best fit for your research. If you are not sure, read the appropriate sections before making your selection.

☐ Life sciences ☒ Behavioural & social sciences ☐ Ecological, evolutionary & environmental sciences

For a reference copy of the document with all sections, see nature.com/documents/nr-reporting-summary-flat.pdf

Behavioural & social sciences study design

All studies must disclose on these points even when the disclosure is negative.

Study description	This study performed a quantitative analysis of de-identified secondary, longitudinal data on child growth.
Research sample	The data analyzed in this study were amassed as part of the Knowledge Integration (ki) initiative of the Bill & Melinda Gates Foundation, which aggregated observations on millions of participants from a global collection of studies on child birth, growth and development. We selected longitudinal cohorts from the database that met five inclusion criteria: 1) conducted in LMICs; 2) enrolled children between birth and age 24 months and measured their length and weight repeatedly over time; 3) did not restrict enrollment to acutely ill children; 4) enrolled at least 200 children; and 5) collected anthropometry measurements at least every 3 months. Thirty-one cohorts met inclusion criteria, including 52,640 children and 412,458 total measurements.
Sampling strategy	Not applicable.
Data collection	Not applicable.
Timing	Included datasets were collected between 1987 and 2017.
Data exclusions	We dropped 859 out of 413,317 measurements (0.2%) from the original datasets because length-for-age Z-scores were unrealistic (> 6 or < –6 Z).
Non-participation	Not applicable.
Randomization	Participants were not randomly assigned.

Reporting for specific materials, systems and methods

We require information from authors about some types of materials, experimental systems and methods used in many studies. Here, indicate whether each material, system or method listed is relevant to your study. If you are not sure if a list item applies to your research, read the appropriate section before selecting a response.

Materials & experimental systems

n/a	Involved in the study	
☒	☐	Antibodies
☒	☐	Eukaryotic cell lines
☒	☐	Palaeontology
☒	☐	Animals and other organisms
☒	☐	Human research participants
☒	☐	Clinical data

Methods

n/a	Involved in the study	
☒	☐	ChIP-seq
☒	☐	Flow cytometry
☒	☐	MRI-based neuroimaging

nature research

Corresponding author(s): Andrew Mertens, Benjamin F. Arnold

Last updated by author(s): Jun 4, 2020

Reporting Summary

Nature Research wishes to improve the reproducibility of the work that we publish. This form provides structure for consistency and transparency in reporting. For further information on Nature Research policies, see Authors & Referees and the Editorial Policy Checklist.

Statistics

For all statistical analyses, confirm that the following items are present in the figure legend, table legend, main text, or Methods section.

n/a	Confirmed	
☐	☒	The exact sample size (n) for each experimental group/condition, given as a discrete number and unit of measurement
☐	☒	A statement on whether measurements were taken from distinct samples or whether the same sample was measured repeatedly
☒	☐	The statistical test(s) used AND whether they are one- or two-sided<br>*Only common tests should be described solely by name; describe more complex techniques in the Methods section.*
☐	☒	A description of all covariates tested
☐	☒	A description of any assumptions or corrections, such as tests of normality and adjustment for multiple comparisons
☐	☒	A full description of the statistical parameters including central tendency (e.g. means) or other basic estimates (e.g. regression coefficient) AND variation (e.g. standard deviation) or associated estimates of uncertainty (e.g. confidence intervals)
☐	☒	For null hypothesis testing, the test statistic (e.g. F, t, r) with confidence intervals, effect sizes, degrees of freedom and P value noted<br>*Give P values as exact values whenever suitable.*
☒	☐	For Bayesian analysis, information on the choice of priors and Markov chain Monte Carlo settings
☐	☒	For hierarchical and complex designs, identification of the appropriate level for tests and full reporting of outcomes
☐	☒	Estimates of effect sizes (e.g. Cohen's d, Pearson's r), indicating how they were calculated

Our web collection on statistics for biologists contains articles on many of the points above.

Software and code

Policy information about availability of computer code

Data collection	This manuscript is a secondary data analysis of existing study data from 31 cohorts and trials, so we were not involved in original data collection.
Data analysis	All analyses were conducted using R statistical software, and scripts that reproduce all analyses are available on Github here: https://github.com/child-growth/ki-longitudinal-growth

For manuscripts utilizing custom algorithms or software that are central to the research but not yet described in published literature, software must be made available to editors/reviewers. We strongly encourage code deposition in a community repository (e.g. GitHub). See the Nature Research guidelines for submitting code & software for further information.

Data

Policy information about availability of data

All manuscripts must include a data availability statement. This statement should provide the following information, where applicable:

- Accession codes, unique identifiers, or web links for publicly available datasets
- A list of figures that have associated raw data
- A description of any restrictions on data availability

The data that support the findings of this study are available from the the original study data contributors upon reasonable request (email contact: amertens@berkeley.edu).

Field-specific reporting

Please select the one below that is the best fit for your research. If you are not sure, read the appropriate sections before making your selection.

☐ Life sciences ☒ Behavioural & social sciences ☐ Ecological, evolutionary & environmental sciences

Behavioural & social sciences study design

All studies must disclose on these points even when the disclosure is negative.

Study description	This study performed a quantitative analysis of de-identified secondary, longitudinal data on child growth.
Research sample	The data analyzed in this study were amassed as part of the Knowledge Integration (ki) initiative of the Bill & Melinda Gates Foundation, which aggregated observations on millions of participants from a global collection of studies on child birth, growth and development. We selected longitudinal cohorts from the database that met five inclusion criteria: 1) conducted in LMICs; 2) enrolled children between birth and age 24 months and measured their length and weight repeatedly over time; 3) did not restrict enrollment to acutely ill children; 4) enrolled at least 200 children; and 5) collected anthropometry measurements at least every 3 months. Thirty-one cohorts met inclusion criteria, including 94,019 children and 645,869 total measurements, and an additional 4 studies including and 14,317 children and 70,659 additional measurements were used in an additional analysis of child mortality.
Sampling strategy	Not applicable.
Data collection	Not applicable.
Timing	Included datasets were collected between 1969 and 2014.
Data exclusions	We dropped 1,332 (0.2%) unrealistic measurements of length-for-age Z-scores (> 6 or < –6 Z), 1,493 (0.2%) measurements of weight-for-age Z-scores (> 6 or < –5 Z), and 1,834 (0.3%) measurements of weight-for-length Z-scores (> 5 or < –5 Z), consistent with WHO recommendations.
Non-participation	Not applicable.
Randomization	Participants were not randomly assigned.

Reporting for specific materials, systems and methods

We require information from authors about some types of materials, experimental systems and methods used in many studies. Here, indicate whether each material, system or method listed is relevant to your study. If you are not sure if a list item applies to your research, read the appropriate section before selecting a response.

Materials & experimental systems

n/a	Involved in the study	
☒	☐	Antibodies
☒	☐	Eukaryotic cell lines
☒	☐	Palaeontology
☒	☐	Animals and other organisms
☒	☐	Human research participants
☒	☐	Clinical data

Methods

n/a	Involved in the study	
☒	☐	ChIP-seq
☒	☐	Flow cytometry
☒	☐	MRI-based neuroimaging
