# Supplementary material for: Author Correction: Early-childhood linear growth faltering in low- and middle-income countries
Source: Nature. 2024 Dec 12;637(8045):E18. doi: 10.1038/s41586-024-08344-6 (PMC11711084; doi:10.1038/s41586-024-08344-6)

nature portfolio

https://doi.org/10.1038/s41586-024-08344-6

Supplementary information

Author Correction: Early-childhood linear growth faltering in low- and middle-income countries

In the format provided by the authors and unedited

Original Figure 3a

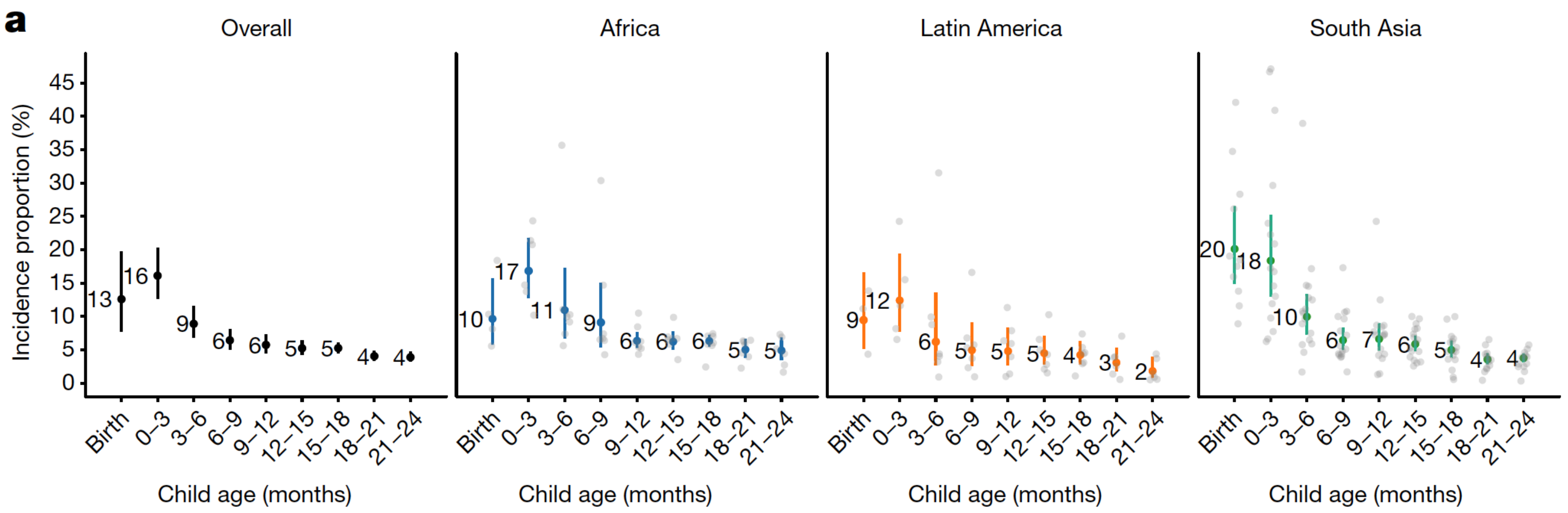

Corrected Figure 3a (coding error corrected)

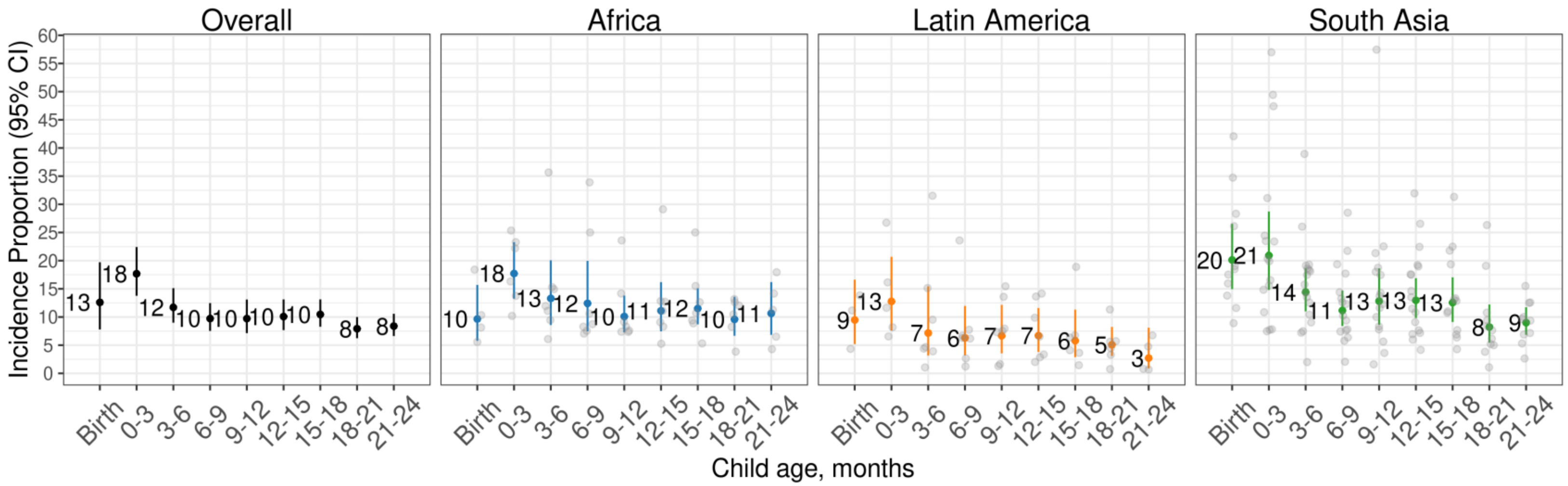

Original Figure 4b

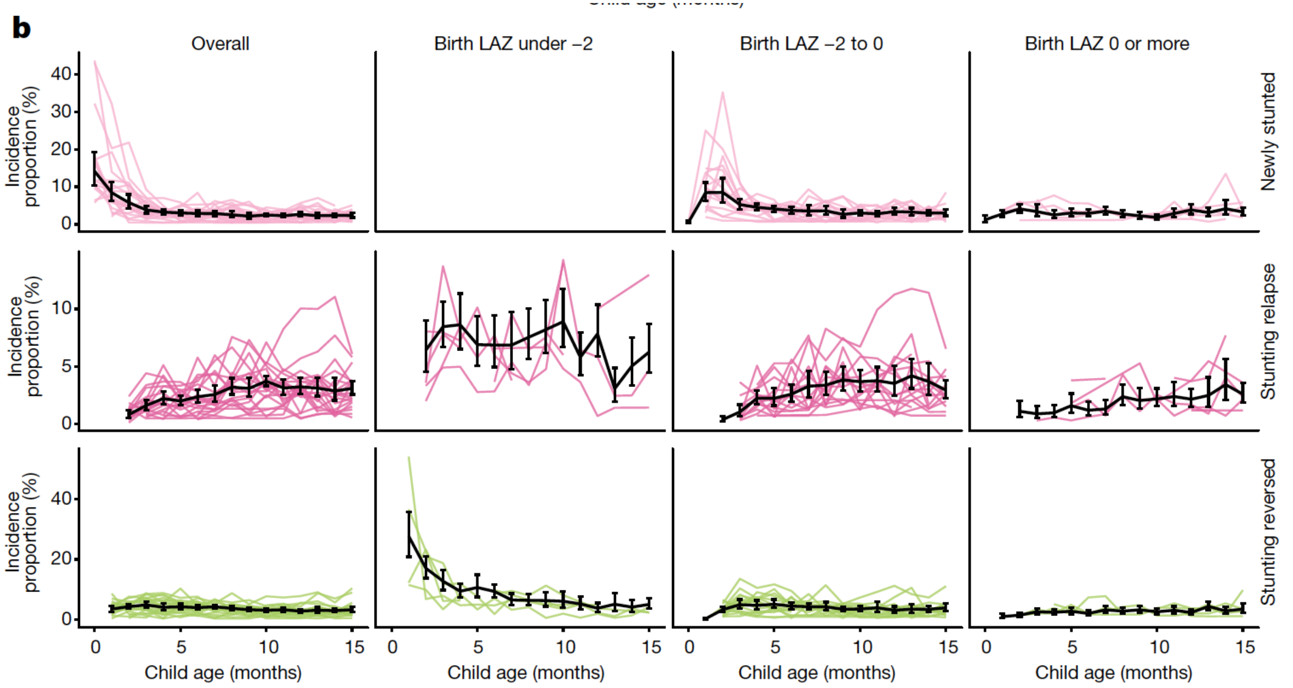

Corrected Figure 4b (labeling error corrected)

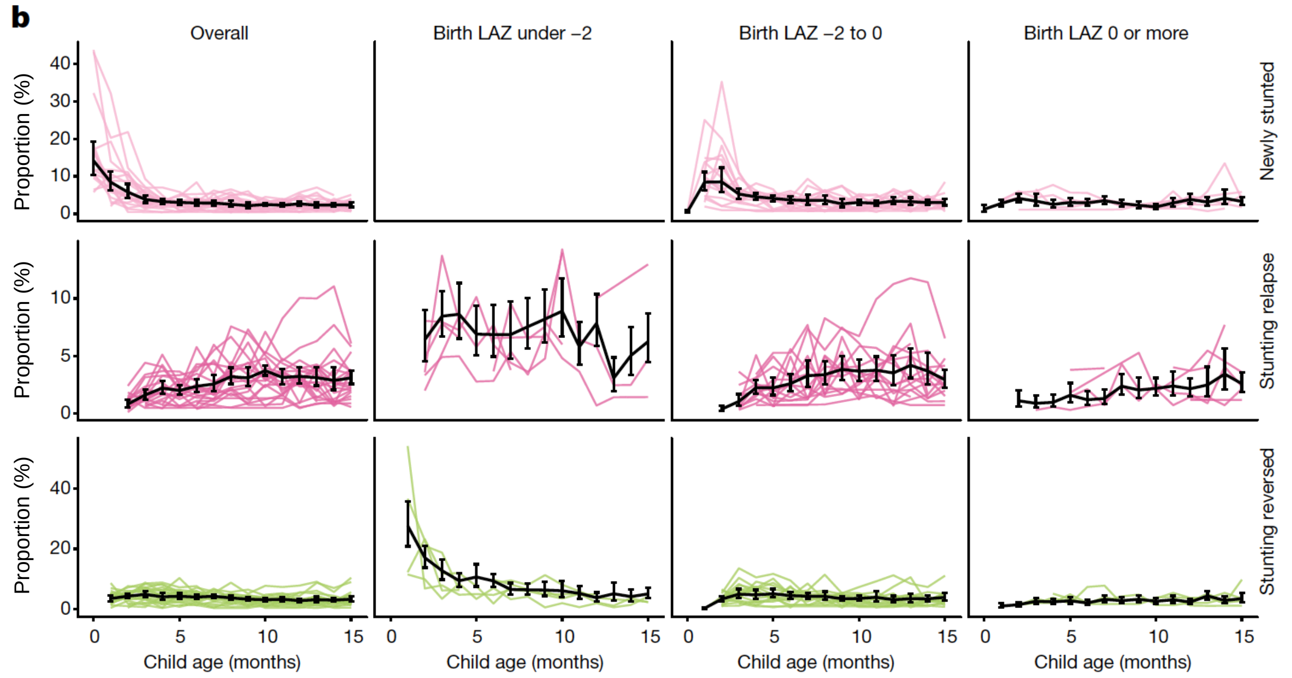

Original Figure ED8

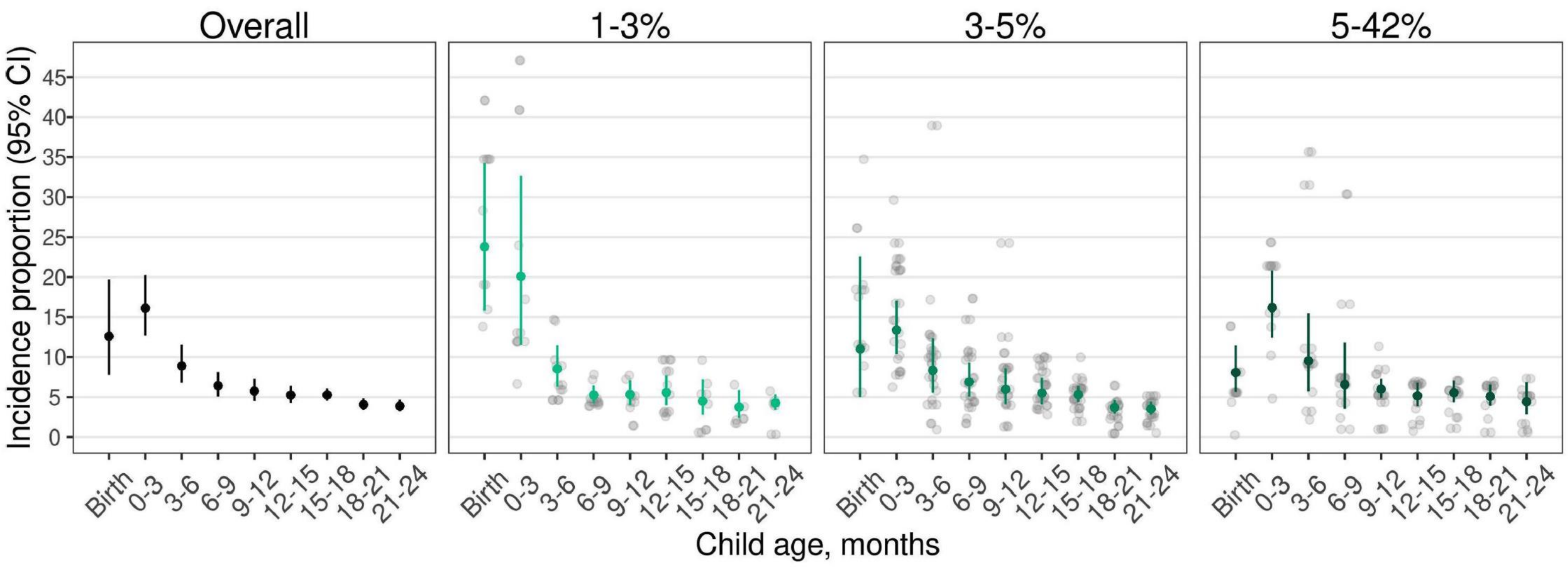

Corrected Figure ED8(coding error corrected)

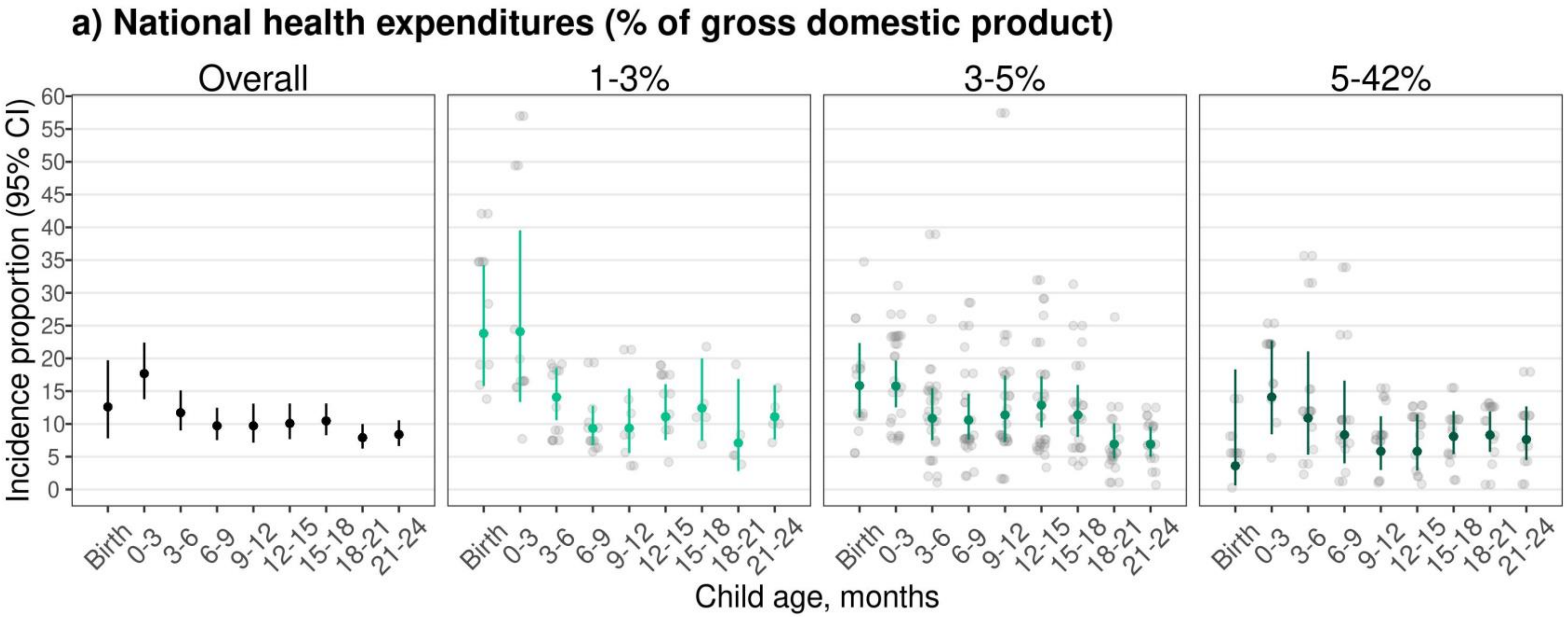

Original Figure ED9

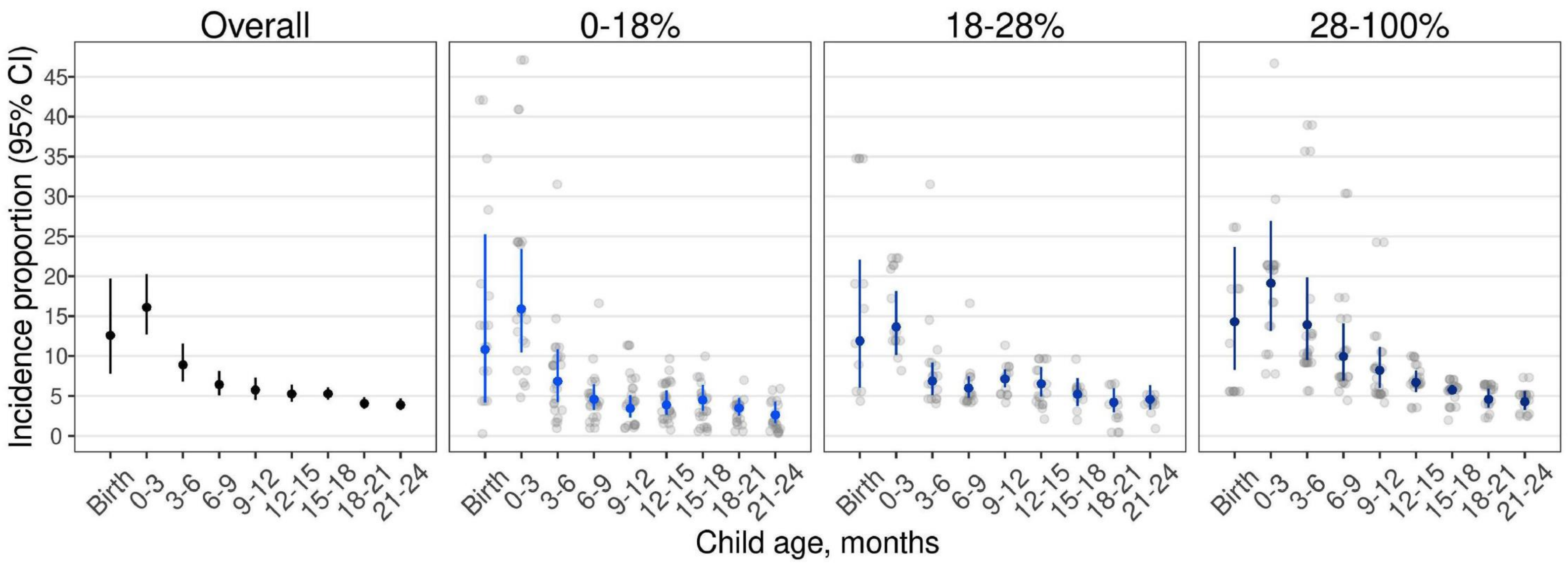

Corrected Figure ED9(coding error corrected)

b) National percentage of individuals living on less than $1.90 per day

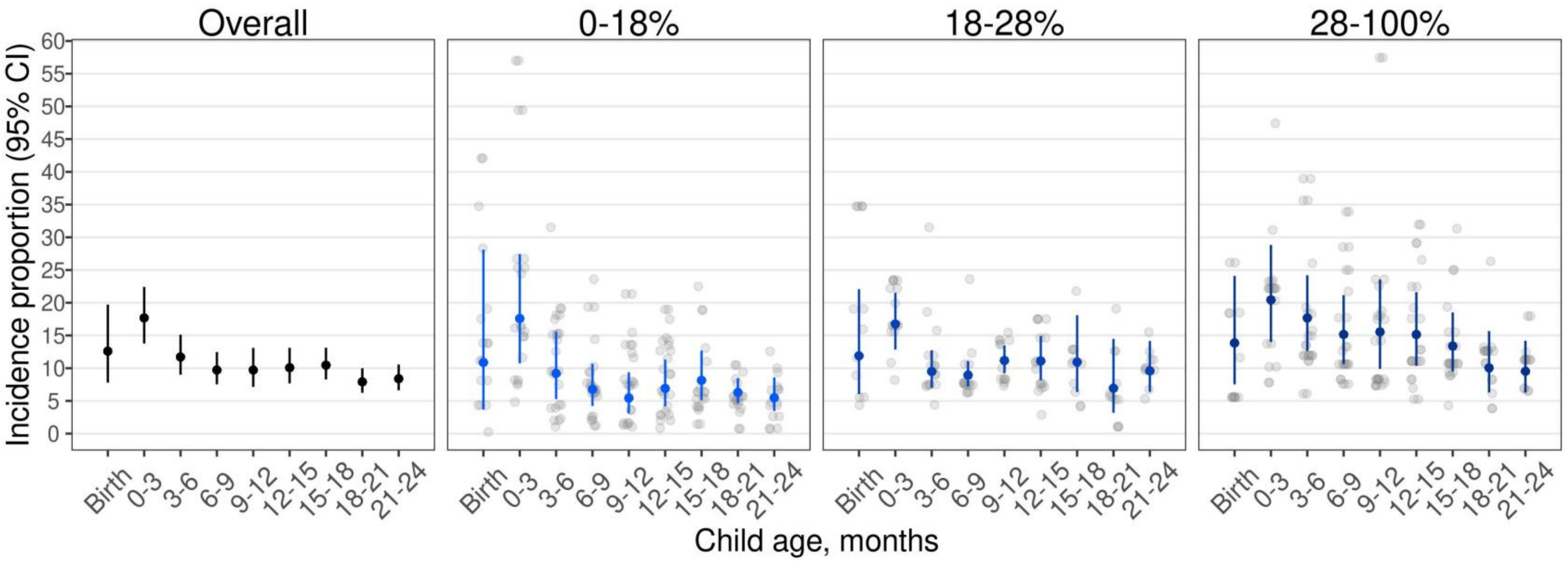

Original Figure ED10

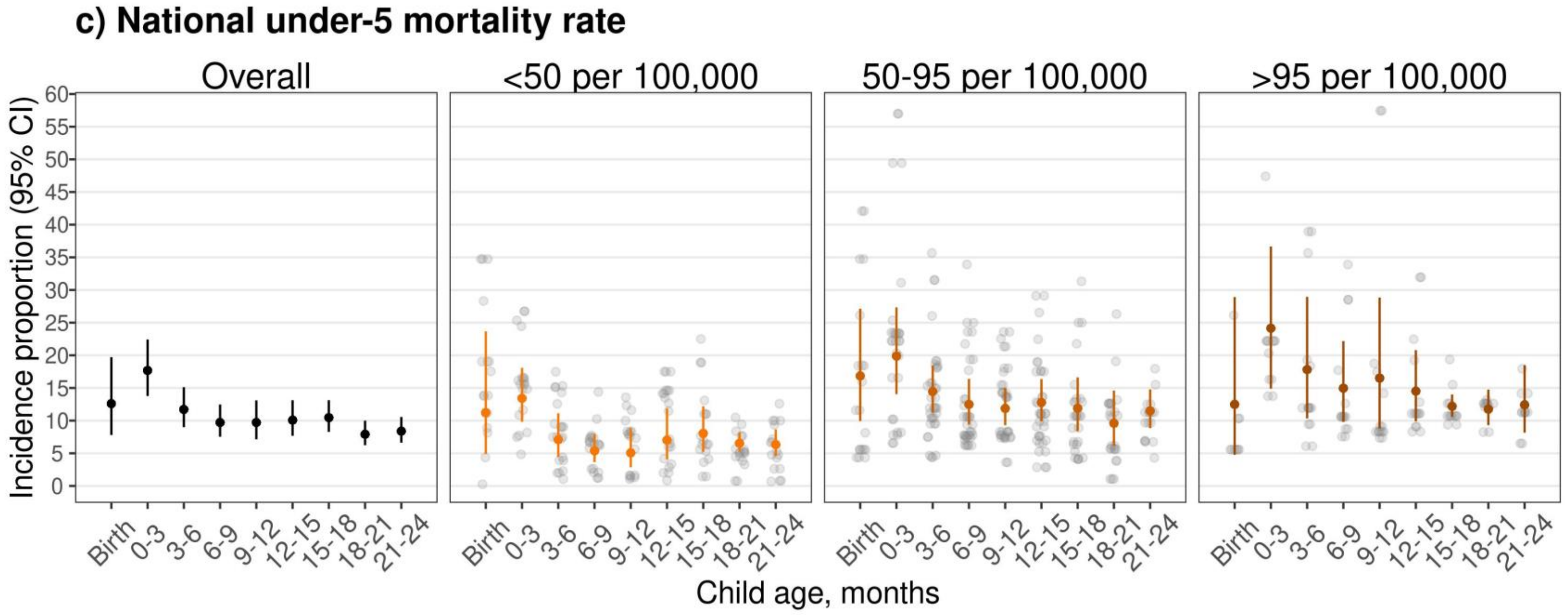

Corrected Figure ED10 (coding error corrected)

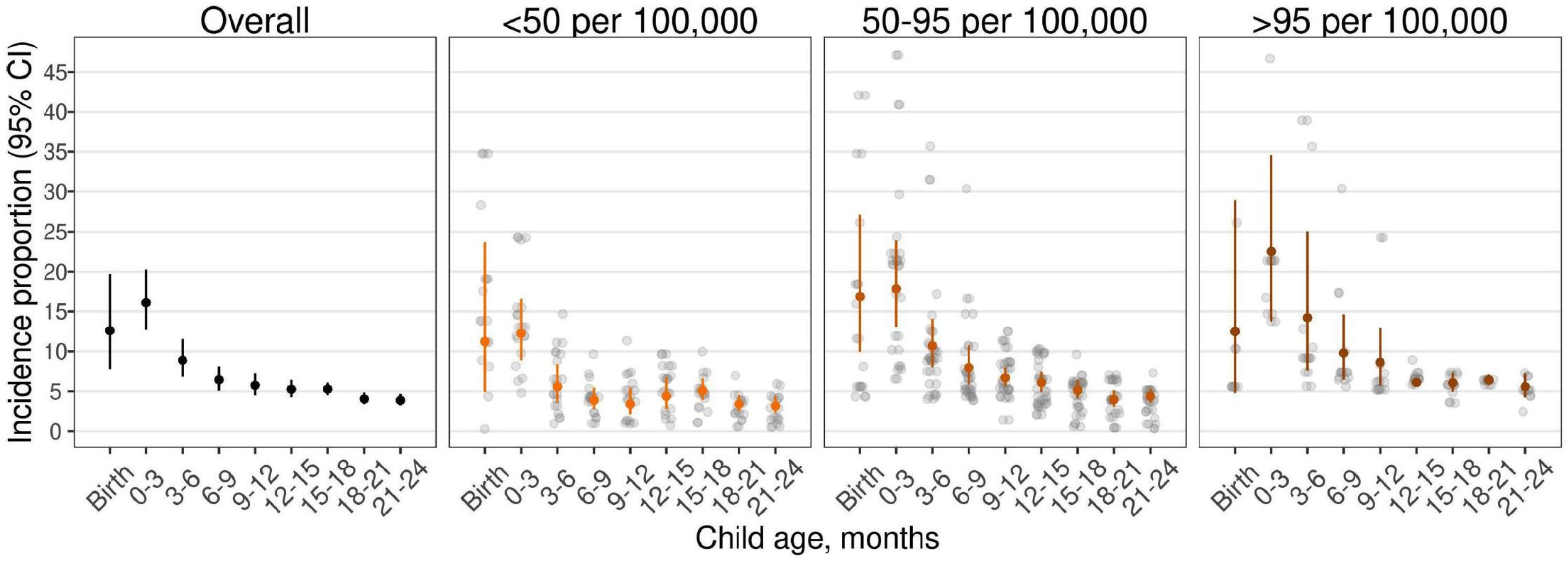

Original Figure ED12

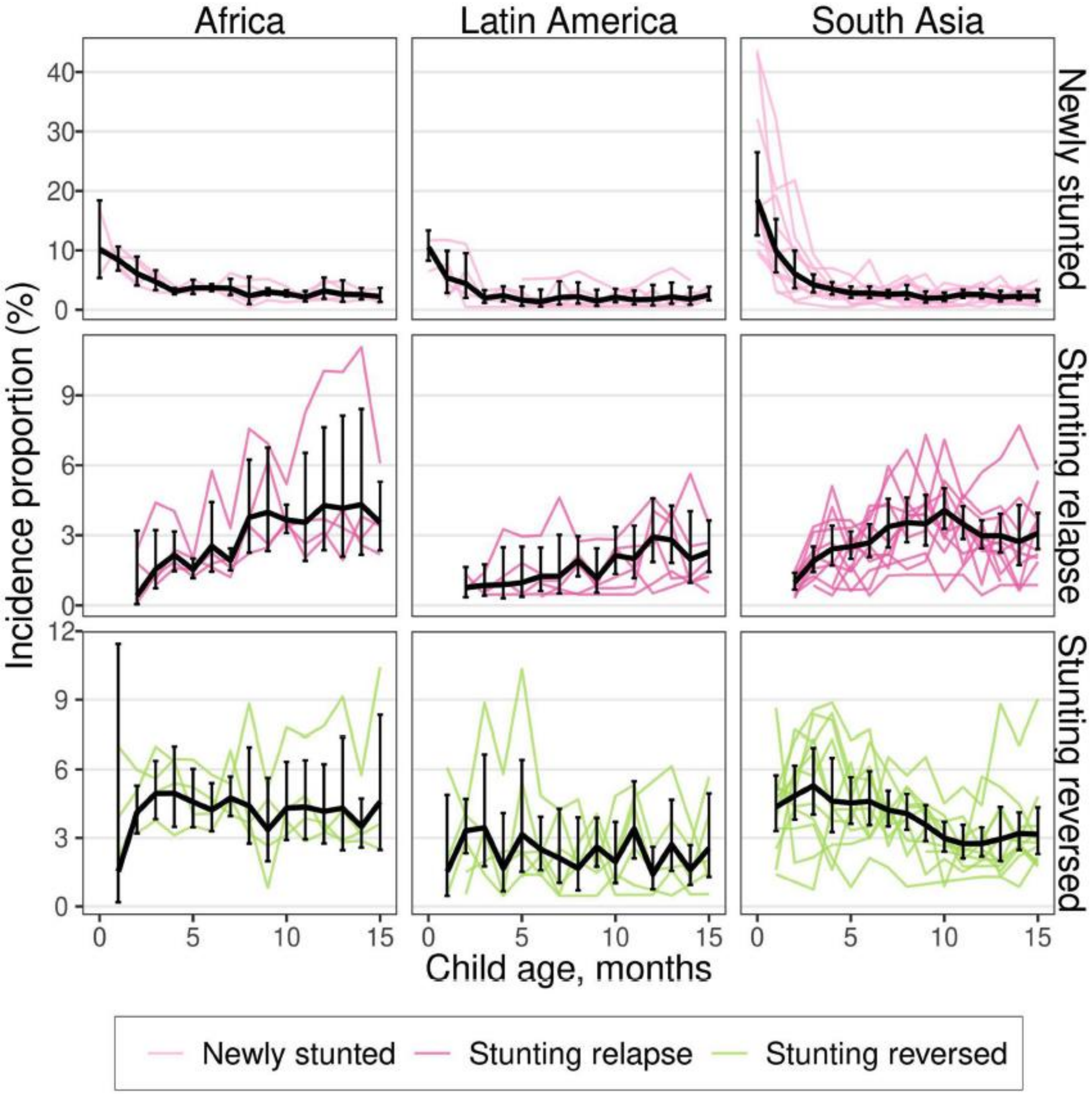

Corrected Figure ED12 (labeling error

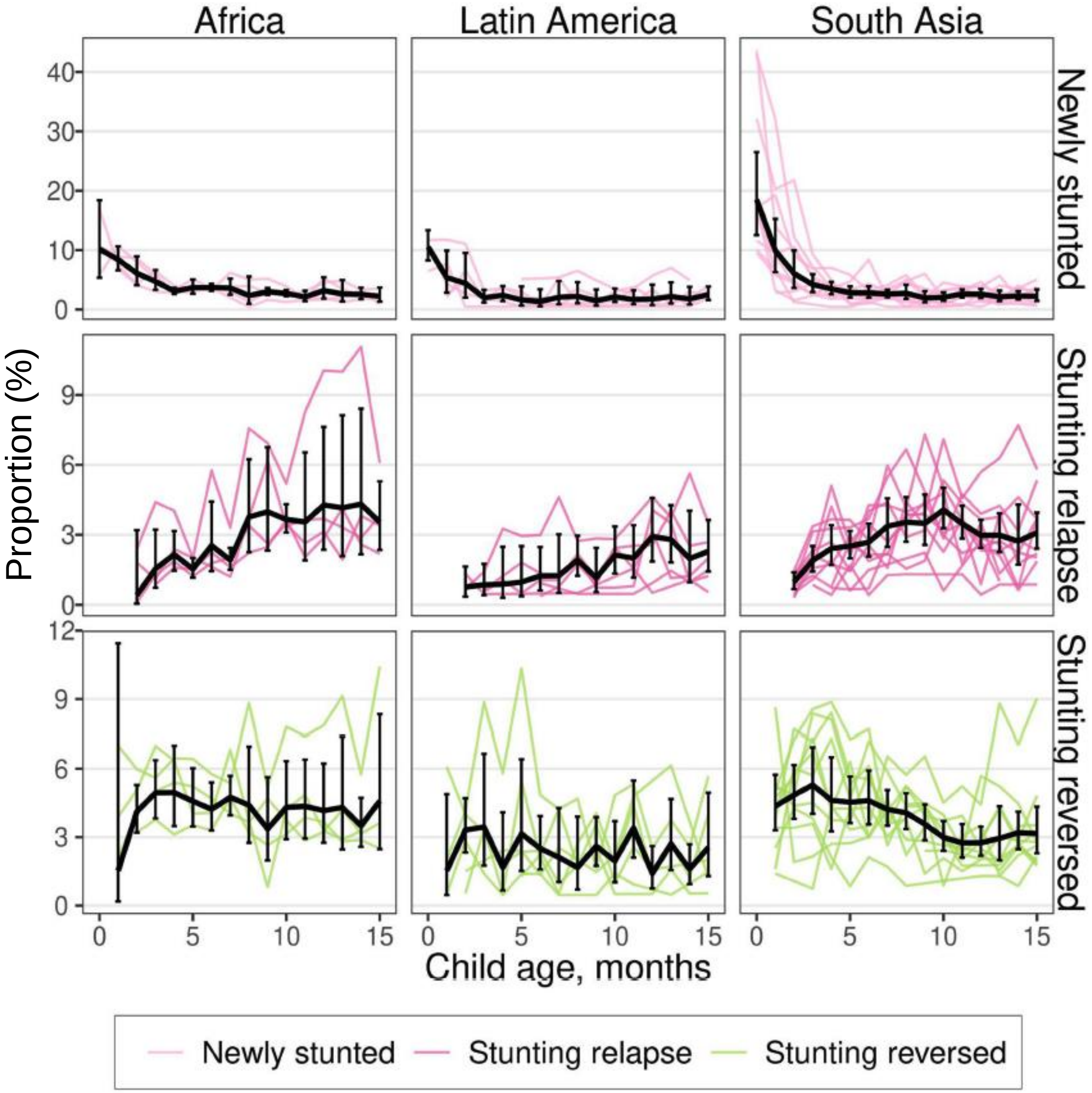

Supplement: Supplementary file 1 — Original and revised Figs. 3a, 4b, Extended Data Figs. 8, 9, 10, 12 [file 41586_2024_8344_MOESM1_ESM.pdf]
